# Mitochondrial fission surveillance is coupled to *Caenorhabditis elegans* DNA and chromosome segregation integrity

**DOI:** 10.1371/journal.pgen.1011678

**Published:** 2025-04-25

**Authors:** Xiaomeng Yang, Ruichen Wei, Fanfan Meng, Dianchen Liu, Xuan Gong, Gary Ruvkun, Wei Wei

**Affiliations:** 1 College of Life Sciences, Capital Normal University, Beijing, China; 2 Department of Molecular Biology, Massachusetts General Hospital, Boston, Massachusetts, United States of America; 3 Department of Genetics, Harvard Medical School, Boston, Massachusetts, United States of America; University of California San Diego, UNITED STATES OF AMERICA

## Abstract

Mitochondrial fission and fusion are tightly regulated to specify mitochondrial abundance, localization, and arrangement during cell division as well as in the diverse differentiated cell types and physiological states. However, the regulatory pathways for such mitochondrial dynamics are less explored than the mitochondrial fission and fusion components. Here we report a large-scale screen for genes that regulate mitochondrial fission. Mitochondrial fission defects cause a characteristic uneven fluorescent pattern in embryos carrying mitochondrial stress reporter genes. Using this uneven activation, we performed RNAi screens that identified 3 kinase genes from a ~ 500-kinase library and another 11 genes from 3,300 random genes that function in mitochondrial fission. Many of these identified genes play roles in chromosome segregation. We found that chromosome missegregation and genome instability lead to dysregulation of mitochondrial fission, possibly independent of DRP-1. ATL-1, the *C. elegans* ATR orthologue, plays a potentially protective role in alleviating the mitochondrial fission defect caused by chromosome missegregation. This establishes a screening paradigm for identifying mitochondrial fission regulators, which reveals the potential role of ATR in surveilling mitochondrial fission to mitigate dysregulation caused by improper chromosome segregation.

## Author summary

Mitochondrial dynamics is critical to the organelle’s functionality. The imbalance of mitochondrial fission and fusion is implicated in a variety of human diseases, including cancer, cardiovascular diseases, metabolic syndromes, and neurodegenerative disorders. Therefore, identifying the genes and mechanisms that regulate mitochondrial dynamics is of paramount importance. Traditionally, studies on mitochondrial dynamics have relied heavily on high-resolution imaging of mitochondrial morphology, which presents challenges for conducting large-scale genetic screens. In this study, we report a readily observable phenotype that is closely associated with defects in mitochondrial fission. Based on this phenotype, we establish a screening paradigm for identifying genes involved in mitochondrial fission. Through two RNA interference screens, we have identified several new genes functioning in chromosome segregation that also play a role in regulating mitochondrial fission. Furthermore, our work reveals a potential new role for the DNA damage sensor kinase ATR in surveilling mitochondrial fission and maintaining mitochondrial function in response to improper chromosome segregation. These findings illuminate a previously unrecognized mechanism through which the nucleus communicates with and regulates mitochondrial activity.

## Introduction

To meet the widely varying energy and metabolism needs of diverse cell types, mitochondria continuously undergo fission and fusion, making them highly dynamic organelles [1–4]. Dysregulation of mitochondrial dynamics, manifesting as abnormal mitochondrial morphology, is evident in a wide range of human diseases including cancer, cardiovascular diseases, metabolic syndromes, and neurodegeneration [5–7]. Disruption of mitochondrial dynamics is a critical factor influencing mitochondrial function; restoring balance in fission-fusion events improves mitochondrial function in several disease models [8–10].

Mitochondrial fusion and fission are mediated by conserved GTPases, including EAT-3/OPA1 and FZO-1/MFN1, which function in the fusion of the inner and outer mitochondrial membrane, respectively [2,3,11], and DRP-1/DRP1, which mediates the fission of mitochondria [12–14]. The cytoskeleton, including actin, tubulin, and their associated motor proteins, also contributes to mitochondrial dynamics, as well as to transporting and localizing mitochondria during fission or fusion [10,15,16]. Actin aids in the constriction at the fission sites and stimulates the division of the organelle [17–20]. Tubulin can affect mitochondrial fission by modulating DRP1 activity [21,22]. High-resolution microscopy can visualize mitochondrial morphology for the discovery of receptor and mediator proteins of mitochondrial fission or fusion in addition to the known GTPase executors [23–29]. However, it is still challenging to identify regulators of mitochondrial dynamics at a genome-wide scale.

As double-membrane organelles, mitochondria broadly crosstalk with other cellular membrane-bound organelles. Our previous work reveals that the lysosome is involved in the control of mitochondrial dynamics through vitamin B12 metabolism [30]. In addition, the nucleus also frequently communicates with mitochondria, regulating mitochondrial dynamics at mitosis, a process in which mitochondrial fission is required for the proper distribution of mitochondria into daughter cells. The regulation of the pro-fission protein DRP1 has been most extensively studied. DRP1 is highly regulated in a cell cycle-specific manner by posttranslational modifications such as phosphorylation, ubiquitylation, and sumoylation [11,31,32]. For example, mammalian Aurora A kinase (AURKA) facilitates DRP1 phosphorylation by CDK1-Cyclin B1 during M-phase, thereby directing DRP1 to the outer mitochondrial membrane to execute fission [26]. Because fission-dependent mitochondrial distribution is a key step in mitosis, we explored whether other mitotic processes, for example, chromosome segregation, regulate mitochondrial fission. Furthermore, there is a need to clarify how the nucleus communicates with mitochondrial dynamics in pathological conditions, such as in cancer cells, which are often characterized by aneuploidy caused by chromosome segregation errors.

The maintenance of mitochondrial homeostasis and function involves cellular pathways that surveil and protect these organelles. Previous studies revealed surveillance pathways for mitochondrial defects arising from electron transport chain (ETC) and oxidative phosphorylation (OXPHOS) dysregulation. These mechanisms primarily encompass mitochondrial repair, drug detoxification, and immune response [33,34]. However, the surveillance of mitochondrial dynamics remains unexplored.

In this study, we found that a mitochondrial fission defect unevenly activates responsive reporters, resulting in a characteristic fluorescent punctate pattern in *C. elegans* embryos. Based on this, we establish a genetic screen for the identification of new genes that regulate mitochondrial fission, whose primary phenotypic screening, unlike the canonical method, does not rely on the high-resolution images of mitochondrial morphology so it is ready to be scaled up to a genome-wide level. From two RNA interference (RNAi) screens with libraries consisting of approximately 500 kinase genes and 3,300 random genes in the *C. elegans* genome, we isolated three kinase genes, including the *AURKA* orthologue *air-1*, as well as 13 other genes, respectively. Inactivation of any of these three kinase genes, along with 11 out of the 13 candidate genes, causes a mitochondrial fission defect. Several of these genes are involved in chromosome segregation as well as in the fission-dependent distribution of mitochondria during mitosis. We found that chromosome segregation errors and genome instability cause a mitochondrial fission defect, potentially independent of DRP-1. Moreover, our findings suggest that ATL-1/ATR and possibly ATM-1/ATM play protective and surveillance roles in the dysregulation of mitochondrial fission induced by genome instability, thereby contributing to the maintenance of mitochondrial function.

## Results

### DRP-1 deficiency causes a characteristic punctate pattern of the mitochondrial stress response gene expression in *C. elegans* embryos

**Fig 1:**
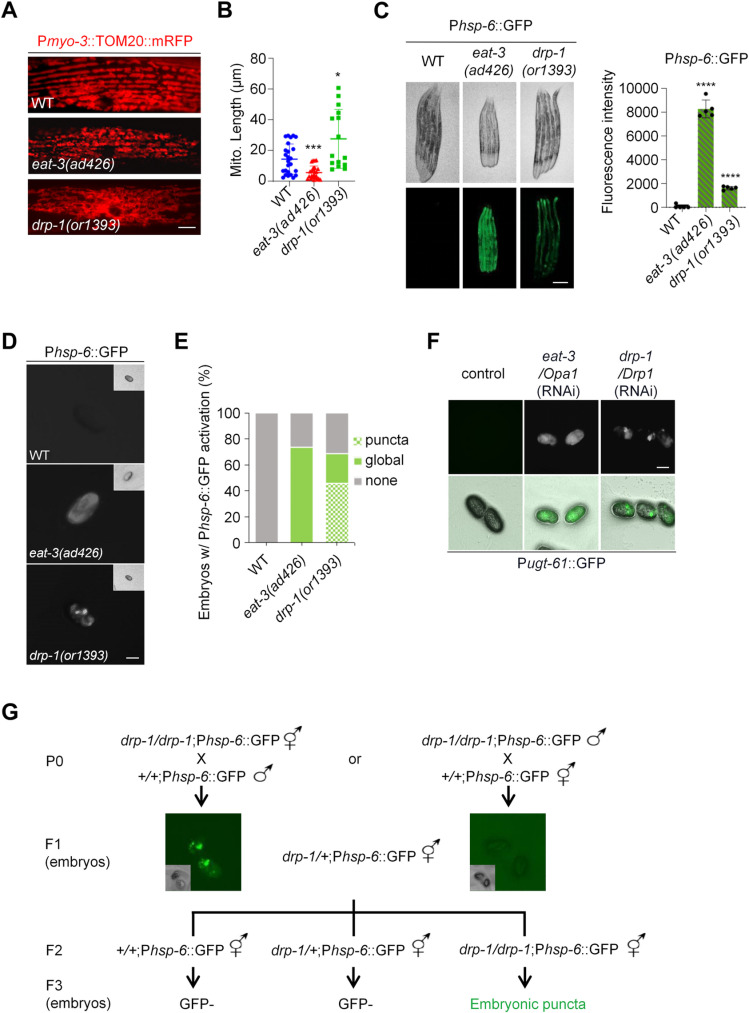
DRP-1 deficiency causes a characteristic punctate pattern of the responsive reporters in *C. elegans* embryos. (A) Mitochondrial morphology in a single body wall muscle cell in wild-type (WT), *eat-3(ad426)*, and *drp-1(or1393)* animals. Scale bar, 5 μm. (B) Mitochondrial lengths in body wall muscles in wild-type (WT), *eat-3(ad426)*, and *drp-1(or1393)* animals. *n* > 15 per group. Median with 95% C. I. Mann-Whitney test. ***P < 0.001, *P < 0.05. (C) P*hsp-6*::GFP expression in WT, *eat-3(ad426)*, and *drp-1(or1393)* adult animals. *n* = 5 per group. Each data point represents the fluorescence intensity measured from a single animal. Mean ± s.d. ****P < 0.0001. Scale bar, 0.25 mm. (D) P*hsp-6*::GFP activation patterns in wild-type, *eat-3(ad426)*, and *drp-1(or1393)* embryos. Scale bar, 25 μm. (E) Percentage of embryos with indicated P*hsp-6*::GFP activation patterns in WT, *eat-3(ad426)*, and *drp-1(or1393)* embryos. *n* > 100 per group. Data represent 3 biological replicates. (F) P*ugt-61*::GFP activation patterns in animals with indicated RNAi treatments. Scale bar, 30 μm. (G) The inheritance of the embryonic P*hsp-6*::GFP puncta in *drp-1(or1393)* mutant.

Disruption of mitochondrial dynamics causes changes in mitochondrial morphology, which can be visualized by fusing mitochondrial proteins with fluorescent proteins and using high-resolution microscopy. We used an mRFP fluorescent protein fused at the N-terminal mitochondrial targeting sequence (MTS) of the outer mitochondrial membrane translocase TOM20 to visualize the mitochondrial morphology in *C. elegans* body wall muscles, where the wild-type mitochondria localize along the muscle sarcomeres, displaying a highly parallel, periodic, and tubular-like morphology (Figs 1A and S1A). The mitochondrial fusion defect caused by the mutation *eat-3(ad426)* caused mitochondrial fragmentation, consistent with previous results [35] (Fig 1A and 1B). Conversely, a glycine-to-glutamic acid (G39E) mutation on the conserved GTPase domain of the pro-fission protein DRP-1 led to a highly tangled and hyperfused mitochondrial morphology, with the swollen and blebbed structures in some regions, indicative of a more severe mitochondrial fission defect [12,30,36] (Fig 1A and 1B). Gene inactivations of *eat-3* and *drp-1* by RNAi also caused similar mitochondrial morphological changes, consistent with their known function in mitochondrial fusion and fission, respectively (S1A Fig).

Mitochondria are surveilled by a series of cellular pathways [33,34]. Disruption of mitochondrial homeostasis activates mitochondrial-specific chaperone HSP-6-mediated unfolded protein response (mtUPR) [37,38]. Mutations in *eat-3* and *drp-1* both robustly induced P*hsp-6*::GFP, mainly in *C. elegans* intestine, with *eat-3(ad426)* having a stronger mtUPR induction (Fig 1C). In embryos, P*hsp-6*::GFP activation was present in 73% of *eat-3(ad426)* and 69% of *drp-1(or1393)* embryos (Fig 1D and 1E). In *eat-3(ad426)* embryos, as in the intestines, the fluorescence signal was distributed generally evenly (Fig 1D). But in approximately half (46%) of the *drp-1(or1393)* fission defective embryos, P*hsp-6*::GFP was activated unevenly in particular blastomeres, with high fluorescence intensity in some regions of the embryos, displaying a characteristic uneven GFP expression pattern (Fig 1D and 1E). However, while mitochondrial defects caused by disruptions of electron transport chain (ETC) and oxidative phosphorylation (OXPHOS) also strongly induced P*hsp-6*::GFP, the activation was uniform across the embryos or the mature intestine (S1B and S1C Fig) and quite distinct from the punctate pattern in the *drp-1(or1393)* fission defective embryos.

To investigate whether the characteristic P*hsp-6*::GFP punctate phenotype in *drp-1(or1393)* embryos is due to mtUPR or DRP-1/mitochondrial fission deficiency, we examined another reporter P*ugt-61*::GFP that also responds to mitochondrial dysfunction [33]. We found that gene inactivation of *drp-1* also caused a similar embryonic punctate pattern of P*ugt-61*::GFP activation, which was distinct from the generally evenly distributed fluorescence observed in *eat-3* RNAi embryos (Fig 1F). These results suggest that the uneven activation of the mitochondrial responsive reporters in embryos is caused by the DRP-1/mitochondrial fission deficiency.

We further investigated some other genes whose orthologues are thought to be involved in mitochondrial fission. FIS1 (fission 1) was identified to be a receptor of Dnm1/DRP1 to mediate mitochondrial fission in budding yeast, although its mammalian orthologue appeared to have little if any function in fission [39–41]. We found that mutations in *fis-1* and *fis-2* (both are the *C. elegans FIS1* orthologues) caused mild mitochondrial elongation defects, whereas loss of *mff-2* (*C. elegans* orthologue of the human *MFF* that encodes another identified DRP1 receptor) [42] caused modest mitochondrial elongation (S1D Fig). However, none of these gene mutations activated P*hsp-6*::GFP in embryos, i.e., no GFP signal or GFP puncta in embryos (S1E Fig). These results suggest that the embryonic P*hsp-6*::GFP punctate pattern observed in embryos of *drp-1* mutant animals may be triggered by modest to severe mitochondrial fission defects.

Mitochondria cannot be formed *de novo* and are inherited maternally. We investigated whether and how the characteristic GFP puncta in *drp-1* fission defective embryos are inherited. We used the *drp-1(or1393)* hermaphrodites to cross with the wild-type males and observed the P*hsp-6*::GFP puncta in the F1 heterozygous embryos, whereas using wild-type hermaphrodites to cross with the *drp-1(or1393)* males generated F1 embryos all GFP inactivated (Fig 1G). The P*hsp-6*::GFP punctate pattern could be observed in the F3 *drp-1* homozygous embryos by either cross (Fig 1G). These results indicate that the characteristic GFP puncta in *drp-1* embryos are due to mitochondrial fission defects.

### Disruption of the cytoskeleton causes mitochondrial fission defects and results in an embryonic punctate pattern of the responsive reporters

**Fig 2:**
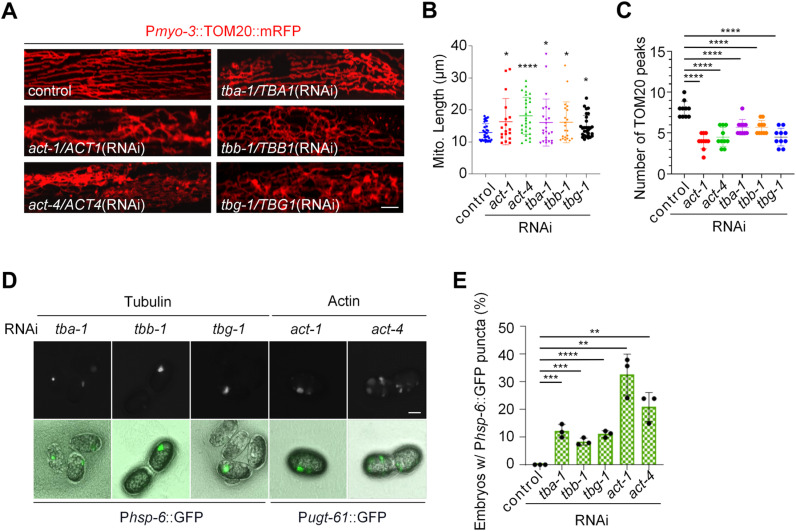
Disruption of the cytoskeleton causes mitochondrial fission defects and results in an embryonic punctate pattern of the responsive reporters. (A) Mitochondrial morphology in a single body wall muscle cell in animals with indicated RNAi treatments. Scale bar, 5 μm. (B) Mitochondrial lengths in animal body wall muscles with indicated RNAi treatments. *n* = 41-50 per group. Each data point represents the mitochondrial length measured from an animal body wall muscle cell. Median with 95% C. I. Mann-Whitney test. ****P < 0.0001, *P < 0.05. (C) TOM20 peak number for the plot profiles of mitochondrial morphology in animals with indicated RNAi treatments. Each data point represents the median TOM20 peak number from the plot profile of mitochondrial morphology in a muscle cell. *n* = 10 per group. Mean ± s.d. ****P < 0.0001. (D) P*hsp-6*::GFP and P*ugt-61*::GFP punctate patterns in embryos caused by disruption of the cytoskeleton. Scale bar, 15 μm. (E) Percentage of embryos with P*hsp-6*::GFP punctate patterns after indicated RNAi treatments. *n* > 120 per group. Data represent 3 biological replicates. Mean ± s.d. ****P < 0.0001, ***P < 0.001, **P < 0.01.

To investigate the effects of the cytoskeleton on *C. elegans* mitochondrial dynamics, we inactivated the actin genes *act-1* or *act-4,* or the tubulin genes *tba-1*, *tbb-1*, or *tbg-1* by RNAi. We found that these gene inactivations all caused highly tangled and hyperfused mitochondria similar to *drp-1* deficiency, suggesting a severe mitochondrial fission defect (Figs 1A, 2A, 2B, and S1A). Mitochondrial length analysis showed that the disruption of these cytoskeleton genes caused increased mitochondrial connectivity (Fig 2B). Due to the mitochondrial fission defect causing tangled and hyperfused mitochondria, with some regions retracting into swollen and blebbed morphologies, the normally wild-type parallel and periodic mitochondrial structure in body wall muscles is disrupted (Figs 1A, 2A, and S1A). To quantify the periodicity of the mitochondrial network, we analyzed the fluorescent plot profiles of multiple cross-sections per mitochondrion using TOM20 peaks [17,30] (S2 Fig). Indeed, the number of TOM20 peaks from the plot profiles of mitochondrial morphologies decreased significantly by RNAi of the cytoskeleton genes (Figs 2C and S2), consistent with the visualized results of mitochondrial morphologies and the analysis of mitochondrial length (Figs 2A-C and S2). Interestingly, we found that disruption of the cytoskeleton by RNAi of the actin or tubulin genes also caused the unique punctate pattern using either the P*hsp-6*::GFP or P*ugt-61*::GFP reporters in 8–33% of embryos (Fig 2D and 2E). Whereas RNAi of the cytoskeleton genes did not significantly activate P*hsp-6*::GFP in the intestine (S3A Fig). Taken together, these results suggest that the characteristic uneven activation of mitochondrial stress response genes in embryos is coupled to a mitochondrial fission defect.

P*hsp-6*::GFP was primarily activated in the intestine of the *drp-1* loss-of-function adult animals (Fig 1C). However, its activation in some regions was discrete, which was more evident in larvae (S3B Fig). We investigated which cells or tissues are responsible for the high induction of responsive genes caused by *drp-1* mutation. We found that P*hsp-6*::GFP was highly activated in muscles and many neural cells and structures in *drp-1(tm1108)* (S3C and S3D Fig). Fission-dependent mitophagy is critical for eliminating damaged mitochondria. Mitochondrial fission defect may compromise mitophagy, leading to the accumulation of damaged mitochondria, which unevenly activates the responsive genes in certain types of cells like muscles and neurons. Because muscles and neurons are tissues that require high mitochondrial activity, they are more prone to generating and accumulating damaged mitochondria. However, RNAi of neither the autophagy-related gene *bec-1/BECN1* nor the mitophagic regulators *pdr-1/PARK2* or *pink-1/PINK1* induced P*hsp-6*::GFP activation in embryos (S3E Fig). Moreover, inactivations of these mitophagy-related genes had negligible effects on mitochondrial morphology (S3F Fig). We cannot exclude the involvement of other mitophagy pathways or additional mitochondrial degradation systems.

### *air-1*, *air-2,* and *tlk-1* are identified as genes whose inactivation causes embryonic P*hsp-6*::GFP puncta and mitochondrial fission defects

**Fig 3:**
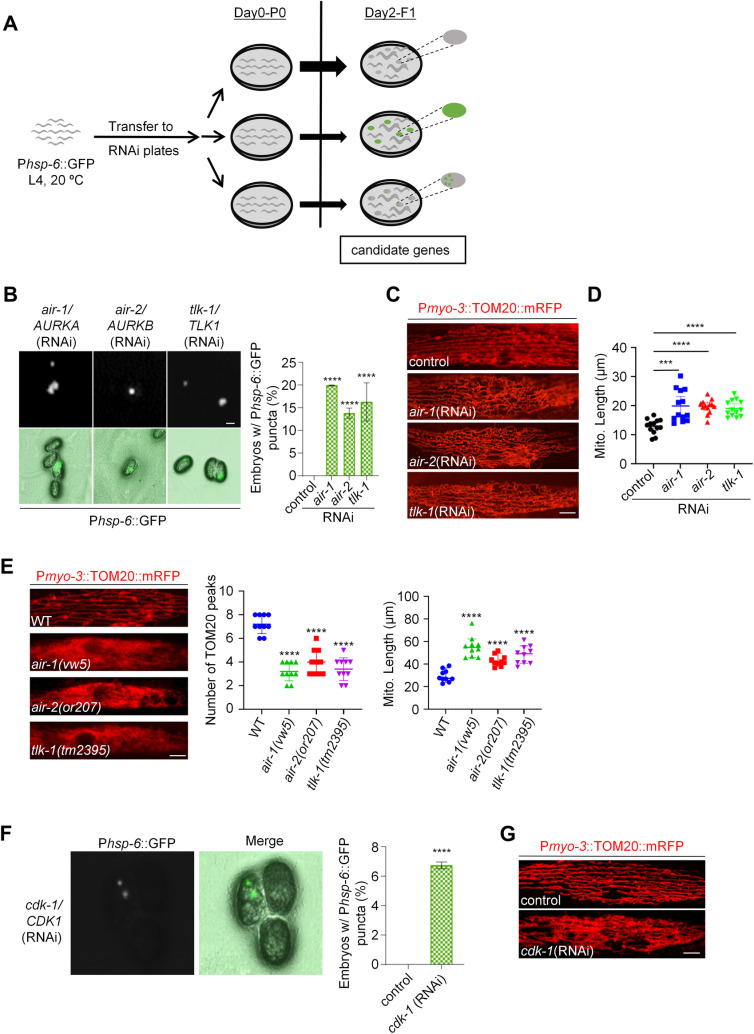
*air-1*, *air-2,* and *tlk-1* are identified as genes whose inactivation causes embryonic P*hsp-6*::GFP puncta and mitochondrial fission defects. (A) Diagram of the RNAi screen workflow for identification of genes whose inactivation results in embryonic P*hsp-6*::GFP puncta. (B) P*hsp-6*::GFP punctate patterns in animal embryos with indicated RNAi treatments. *n* > 120 per group. Data represent 3 biological replicates. Mean ± s.d. ****P < 0.0001. Scale bar, 20 μm. (C) Mitochondrial morphology in a single body wall muscle cell in animals with indicated RNAi treatments. Scale bar, 5 μm. (D) Mitochondrial lengths in animal body wall muscles with indicated RNAi treatments. *n* = 13 per group. Each data point represents the mitochondrial length measured from an animal body wall muscle cell. Median with 95% C. I. Mann-Whitney test. ****P < 0.0001, ***P < 0.001. (E) Mitochondrial morphology in a single body wall muscle cell (left), TOM20 peak number for the plot profiles of mitochondrial morphology (middle, Mean ± s.d.), and mitochondrial lengths body wall muscles (right, Median with 95% C. I. Mann-Whitney test) in indicated animals. ****P < 0.0001. Scale bar, 5 μm. (F) P*hsp-6*::GFP punctate patterns in animal embryos with *cdk-1* RNAi treatment. *n* > 100 per group. Data represent 3 biological replicates. Mean ± s.d. ****P < 0.0001. (G) Mitochondrial morphology in a single body wall muscle cell in animals with indicated RNAi treatments. Scale bar, 5 μm.

We used the uneven activation pattern of responsive gene expression caused by fission defects to identify additional genes involved in mitochondrial fission. We first performed a pilot screen using an RNAi sub-library of approximately 500 *C. elegans* kinase genes (S1 Data), as many kinases regulate cell division, a key feature of the early embryos that unevenly activate *hsp-6* if mitochondrial fission is defective (Fig 3A). From this screen, we identified 3 gene inactivations that cause the characteristic P*hsp-6*::GFP punctate pattern in 14–20% of the embryos (Fig 3B). These include the Aurora A kinase *AURKA* orthologue *air-1*, the Aurora B kinase *AURKB* orthologue *air-2*, and the Tousled-Like Kinase 2 (*TLK2*) orthologue *tlk-1* (Fig 3B).

We further investigated the effects of these three kinase genes on mitochondrial dynamics. To align with the workflow of the screening process (Fig 3A), RNA interference was performed on L4 parental animals and continued into the offspring, after which mitochondrial morphology was assessed in the adult progeny. We found that RNAi of *air-1* caused a fission defect, leading to a highly elongated and hyperfused mitochondrial morphology in body wall muscles (Fig 3C and 3D). This observation is consistent with the function of its human orthologue, *AURKA,* in mitochondrial fission [26]. Similarly, RNAi of both *air-2* and *tlk-1* caused mitochondrial hyperfusion and elongation (Fig 3C and 3D). Furthermore, genetic mutations in *air-1*, *air-2*, and *tlk-1* all caused similar mitochondrial hyperfusion and elongation (Fig 3E). These results suggest that the three identified kinase genes play roles in mitochondrial fission.

AURKA functions in mitochondrial fission by facilitating DRP1 phosphorylation by CDK1 in humans [26]. We then rechecked the *CDK1* orthologue *cdk-1*, which is also included in the kinase sub-library, and found that RNAi of *cdk-1* caused the characteristic P*hsp-6*::GFP puncta in 7% of embryos (Fig 3F). As expected, RNAi of *cdk-1* caused mitochondrial elongation and hyperfusion (Fig 3G), indicating that CDK-1, like its human orthologue, functions in mitochondrial fission. The fact that CDK-1 and possibly other fission regulatory kinases were not identified from the RNAi screen may be due to the high rate of false negatives in a large-scale genetic screen. Taken together, these results indicate that the embryonic GFP punctate pattern of the responsive reporters is tightly coupled to a mitochondrial fission defect.

### 11 new genes that regulate mitochondrial fission

**Fig 4:**
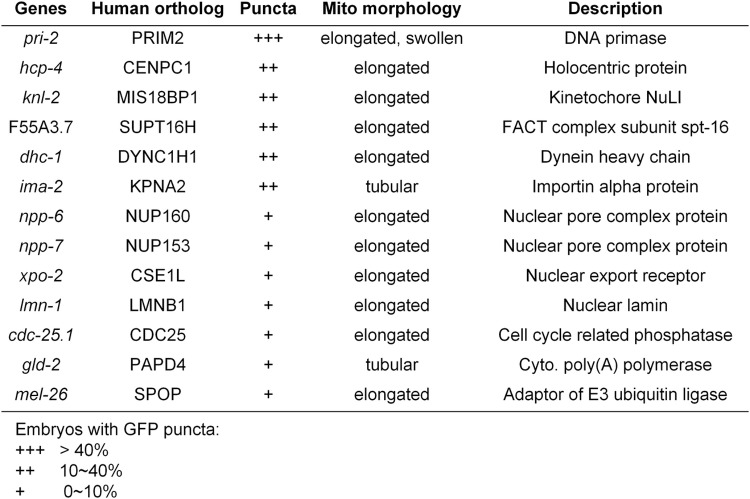
Hits identified from the RNAi screen. Screening 3,300 random genes in the *C. elegans* genome identifies 13 candidates whose gene inactivation results in embryonic P*hsp-6*::GFP puncta.

**Fig 5:**
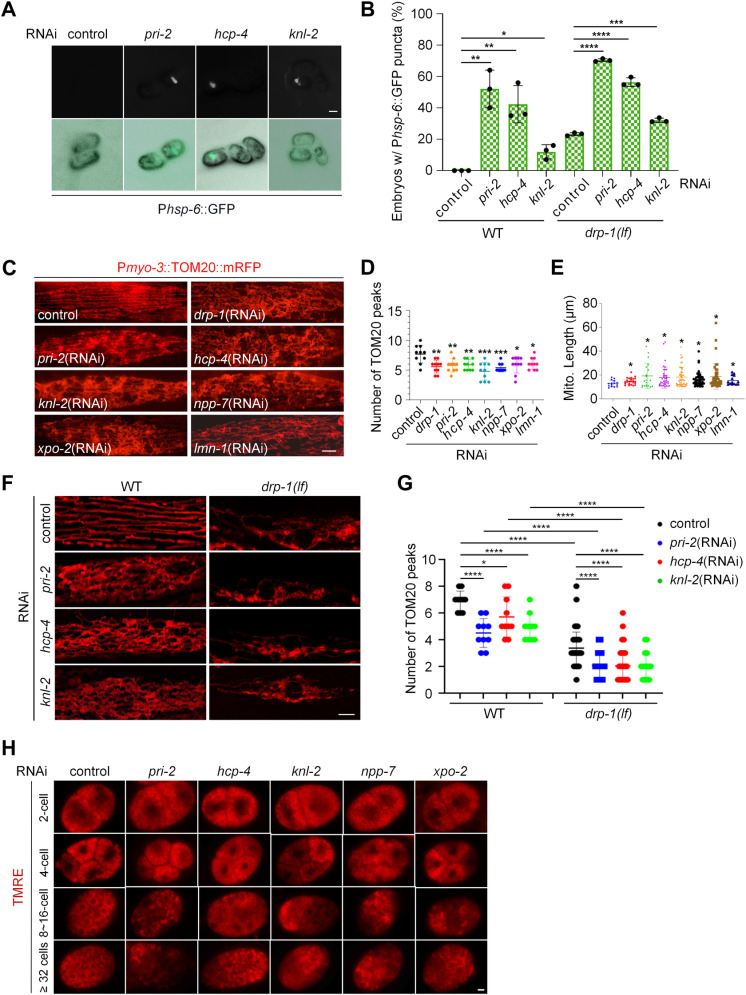
11 genes are identified to be involved in mitochondrial fission. (A) P*hsp-6*::GFP punctate patterns in animal embryos with indicated RNAi treatments. Scale bar, 20 μm. (B) Percentage of embryos with P*hsp-6*::GFP punctate patterns in wild-type (WT) and *drp-1 loss-of-function (lf)* animals after indicated RNAi treatments. *n* > 150 per group. Data represent 3 biological replicates. Mean ± s.d. ****P < 0.0001, ***P < 0.001, **P < 0.01, *P < 0.05. (C) Mitochondrial morphology in a single body wall muscle cell in animals with indicated RNAi treatments. Scale bar, 5 μm. (D) TOM20 peak number for the plot profiles of mitochondrial morphology in animals with indicated RNAi treatments. Each data point represents the median TOM20 peak number from the plot profile of mitochondrial morphology in a muscle cell. *n* = 10 per group. Mean ± s.d. ***P < 0.001, **P < 0.01, *P < 0.05. (E) Mitochondrial lengths in animal body wall muscles with indicated RNAi treatments. *n* = 15–25 per group. Each data point represents the mitochondrial length measured from an animal body wall muscle cell. Median with 95% C. I. Mann-Whitney test. *P < 0.05. (F) Mitochondrial morphology in a single body wall muscle cell in wild-type (WT) and *drp-1(lf)* animals after indicated RNAi treatments. Scale bar, 5 μm. (G) TOM20 peak number for the plot profiles of mitochondrial morphology in wild-type (WT) and *drp-1(lf)* animals with indicated RNAi treatments. Each data point represents the median TOM20 peak number from the plot profile of mitochondrial morphology in a muscle cell. *n* = 10 per group. Mean ± s.d. ****P < 0.0001, *P < 0.05. (H) Mitochondrial distribution in early embryos with indicated RNAi treatments. Mitochondria were indicated by TMRE. Scale bar, 5 μm.

We expanded our RNAi screen to investigate 3,300 random genes from the *C. elegans* genome on Chromosome I and isolated 13 additional gene inactivations that cause P*hsp-6*::GFP puncta in embryos (Figs 4, 5A and 5B, and S2 Data). Similar to the cytoskeleton dysregulation, RNAi of these genes barely induced P*hsp-6*::GFP at later developmental times in the mature intestine (S3A and S4A Figs). It is worth noting that all 13 isolated genes are conserved in mammals, as might be expected for such a universal feature of mitochondrial dynamics (Fig 4).

We further investigated the effects of these 13 gene inactivations on mitochondrial dynamics. RNAi targeting 11 out of the 13 genes caused a highly tangled and hyperfused mitochondrial morphology in body wall muscle cells, similar to that caused by *drp-1* deficiency (Figs 1A and 5C), suggesting that these 11 genes are involved in mitochondrial fission. Hereafter, we will refer to these 11 genes as the “identified genes”. In the following experiments, we will primarily assess several top candidates, including *pri-2*, *hcp-4*, and *knl-2*. Particularly, RNAi of *pri-2* caused the highest percentage of punctate P*hsp-6*::GFP induction, and also caused swollen and blebbed mitochondria in some regions very similar to *drp-1* deficiency, suggesting a severe mitochondrial fission defect (Figs 4 and 5C). RNAi of the newly identified genes reduced TOM20 peaks from the plot profiles of mitochondrial morphologies and led to an increase in mitochondrial lengths (Fig 5D and 5E). Furthermore, the hyperfused and elongated mitochondrial morphology was observed in several available genetic mutants, including *hcp-4(ok1057)*, *knl-2(tm7587)*, and *lmn-1(tm1502)* (S4B Fig), consistent with the results obtained from RNAi experiments. In addition, we investigated the mitochondrial morphology in the mature animal intestine using a reporter expressing GFP in the intestinal mitochondria (P*ges-1*::mitoGFP). RNAi of *pri-2* caused mitochondrial elongation and hyperfusion in the intestine (S4C Fig), consistent with the results in body wall muscles. Similar results were observed following RNAi of several other identified genes, including *hcp-4*, *knl-2*, and *npp-7* (S4C Fig). Dysregulation of mitochondrial dynamics has a direct impact on the functionality of these organelles. Indeed, RNAi of the identified genes caused a loss of mitochondrial membrane potential, as revealed by the Tetramethylrhodamine ethyl ester (TMRE) staining (S4D Fig). Moreover, utilizing an *in vivo* YFP-based hydrogen peroxide sensor HyPer reporter [43], we found that RNAi of many identified genes caused elevated cellular reactive oxygen species (ROS) levels compared to the wild type (S4E Fig), indicating a disturbance in mitochondrial function. Based on these results, it can be inferred that the identified genes play roles in mitochondrial fission.

Additionally, these genes involved in mitochondrial fission appear to function through a pathway that may be independent of *drp-1*, because RNAi of *pri-2*, *hcp-4*, and *knl-2* in a *drp-1* loss-of-function background, *drp-1(tm1108)*, showed an additive effect on the embryonic P*hsp-6*::GFP punctate phenotype (Figs 5B and S4G). The *drp-1(tm1108)* allele harbors approximately 400 bp of insertions and deletions within crucial dynamin domains, resulting in a frame-shift mutation and the introduction of an early stop codon. Moreover, a previous study was unable to detect DRP-1 protein expression in the *drp-1(tm1108)* allele [44]. Therefore, it is probable that *drp-1(tm1108)* represents a null allele. Moreover, RNAi of *pri-2*, *hcp-4*, and *knl-2* in the *drp-1* loss-of-function background exacerbated mitochondrial fission defects, as observed through visualization of mitochondrial morphology and analysis of TOM20 peaks from the plot profiles of mitochondrial morphologies (Fig 5F and 5G). Indeed, previous findings have provided evidence for mitochondrial fission by a mechanism independent of DRP1 [45,46]. This result suggests that the pathway(s) of the identified genes acting in mitochondrial fission may be different from the well-studied mechanisms through regulating DRP1 activity by posttranslational modification.

### The newly identified mitochondrial fission genes affect fission-dependent mitochondrial distribution at mitosis

Mitochondrial fission plays a key role in cell division during mitosis. During this process, mitochondria undergo fragmentation through fission and are subsequently partitioned, often but not always, evenly into daughter cells for proper inheritance [45,47,48]. We investigated whether the newly identified mitochondrial fission genes affect the fission-dependent mitochondrial distribution during *C. elegans* embryogenesis when rapid and coordinated cell division is crucial [49]. Using TMRE to visualize the mitochondria in embryos, we found that RNAi of *pri-2*, as well as *hcp-4*, *knl-2*, *npp-7*, and *xpo-2*, caused an uneven and abnormal distribution of mitochondria into the blastomeres, beginning as early as the 4-cell stage (Fig 5H). Similar results were observed with the use of Nonyl acridine orange (NAO), a fluorescent dye that stains mitochondria independently of mitochondrial membrane potential, in embryos subjected to RNAi targeting *pri-2*, *hcp-4*, and *knl-2* (S4F Fig). Based on these findings, we suggest that these genes play a role in mitochondrial fission during mitosis.

### Inactivation of the new mitochondrial fission genes causes chromosome segregation defects

**Fig 6:**
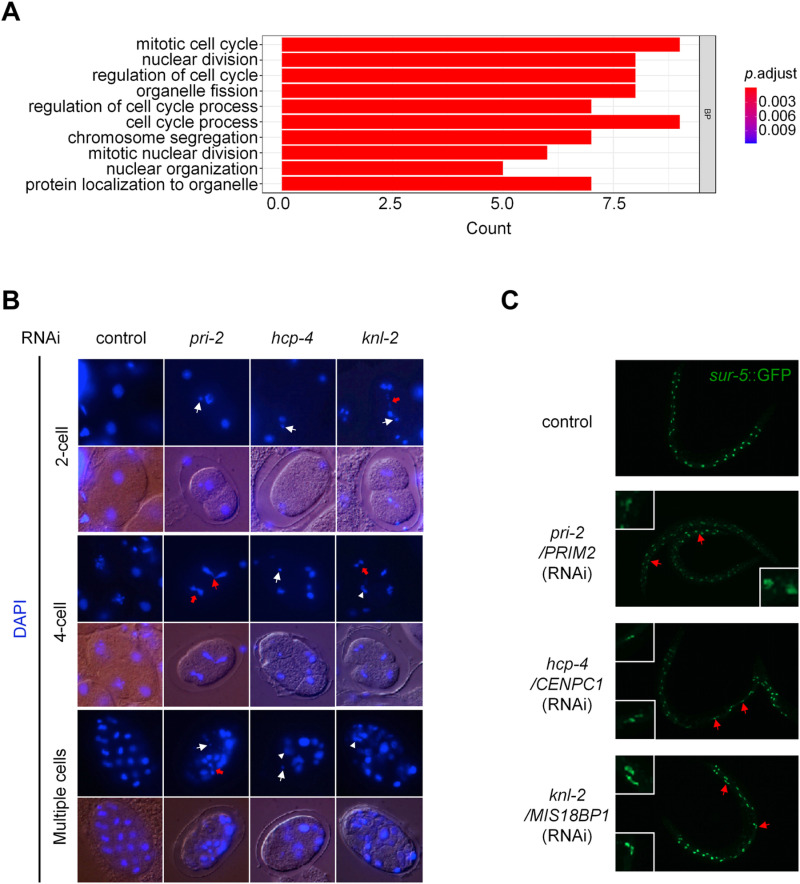
Inactivation of the identified genes causes improper chromosome segregation. (A) Gene Ontology (GO) analysis of the identified genes. (B) Chromosome segregation errors caused by RNAi of *pri-2*, *hcp-4*, and *knl-2*. The nuclei were stained with DAPI. Abnormalities emerged beginning with the first nuclear divisions. Representative abnormalities included but were not limited to micronuclei (white straight arrow), chromosome bridges (red arrow), “cross-eyed” nuclei morphology (red straight arrow), and uneven nuclei distribution (white triangle). (C) Postembryonic cell divisions in animal intestines shown by *sur-5*::GFP. Arrows show abnormal karyotypes in animals with indicated RNAi treatments.

The human orthologues of many identified genes are known to function in several aspects of chromosome segregation (Fig 4) [50–53]. In addition, the Gene Ontology (GO) analysis of all identified genes indicated a significant enrichment in the processes of mitosis and the cell cycle. Among these processes, chromosome segregation stands out as a particularly enriched biological event (Fig 6A), which is crucial for the accurate distribution of genetic material during cell division. We found that RNAi of *pri-2*, *hcp-4*, and *knl-2* all lead to chromosome segregation errors starting from the first cell division during embryogenesis, resulting in micronuclei, chromosome bridges, the “cross-eyed” nuclei morphology, uneven nuclei distribution, etc., in daughter cells (Fig 6B).

To investigate the effects of these identified genes on chromosome segregation in somatic cells, we used a *sur-5*::GFP translational fusion reporter that marks the *C. elegans* intestinal nuclei to monitor the postembryonic cell divisions [54,55]. *C. elegans* hatches with 20 mononucleate intestinal cells at the first larval (L1) stage. During the late L1 stage, 8–12 of the intestinal nuclei duplicate, and the so-called karyokinesis without cytokinesis occurs, resulting in several binucleate intestinal cells [56]. We found that RNAi of *pri-2*, *hcp-4*, and *knl-2* all caused a high frequency of aberrant karyotypes compared to the control (Fig 6C), suggesting that these genes affect chromosome segregation in somatic cells as well.

Improper chromosome segregation on the sex chromosome causes X chromosome non-disjunction, which can lead to loss of the X chromosome during gametogenesis, producing animals with XO genotypes that develop into males, arising in the normal wild-type hermaphrodites (XX) populations [57]. A transcriptionally fused GFP expression reporter driven by a male-specific promoter, P*xol-1*::GFP, can be used to readily detect male embryos *in utero* [58]. We found that RNAi of many of the isolated genes (11 out of 17) induced this male indicator reporter, suggesting a high frequency of chromosome segregation errors caused by these gene inactivations (S5A Fig). Moreover, severe chromosome segregation defects on autosomes increase embryonic lethality [57,59]. Indeed, we found that inactivations of the isolated genes all resulted in high penetrance of embryonic lethality (S5B Fig). Overall, disruption of the identified genes causes improper chromosome segregation.

### Genome instability causes a mitochondrial fission defect that is relieved by ATR

**Fig 7:**
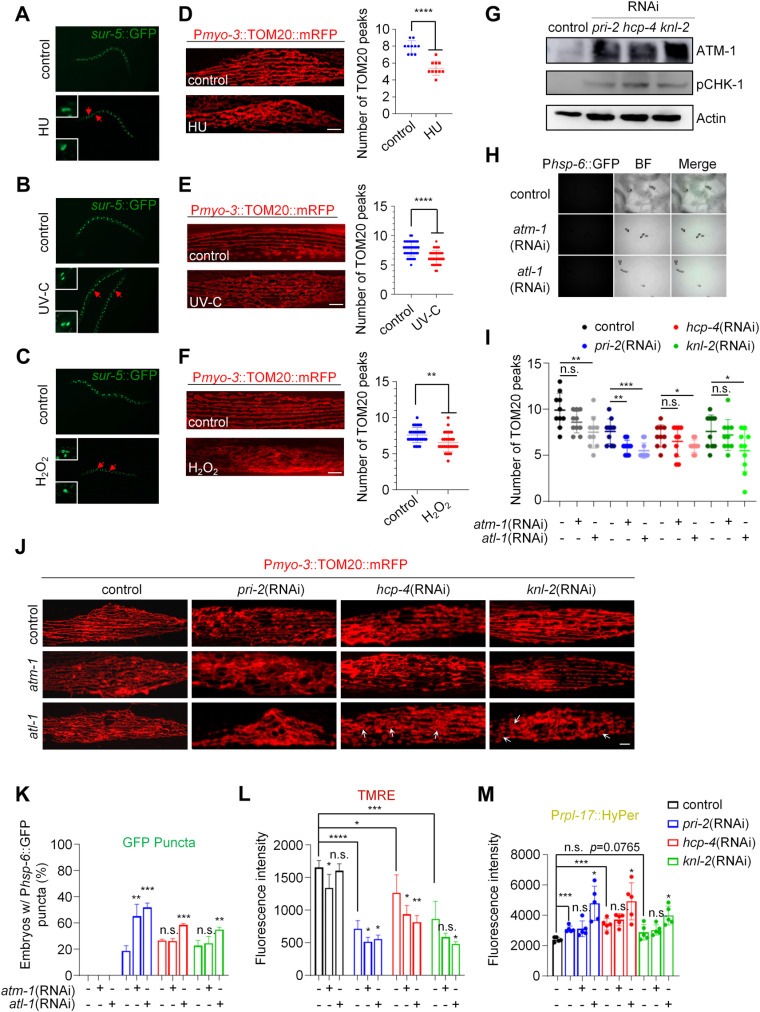
Genome instability causes a mitochondrial fission defect that is relieved by ATR. (A-C) Postembryonic cell divisions in animal intestines shown by *sur-5*::GFP. Arrows show abnormal karyotypes in animals treated with 1 mM HU (A), UV-C (B), and hydrogen peroxide (C). (D-F) Mitochondrial morphology in a single body wall muscle cell in animals treated with 1 mM HU (D), UV-C (E), and hydrogen peroxide (F). TOM20 peak number for the plot profiles of mitochondrial morphology in animals treated with 1 mM HU (D), UV-C (E), and hydrogen peroxide (F). Each data point represents the median TOM20 peak number from the plot profile of mitochondrial morphology in a muscle cell. *n* > 10 per group. Mean ± s.d. ****P < 0.0001, **P < 0.01. Scale bar, 5 μm. (G) Immunoblots of lysates from animals with indicated treatment. (H) P*hsp-6*::GFP expression in animal embryos with indicated RNAi treatments. (I) TOM20 peak number for the plot profiles of mitochondrial morphology in animals with indicated RNAi treatments. Each data point represents the median TOM20 peak number from the plot profile of mitochondrial morphology in a muscle cell. *n* = 10 per group. Mean ± s.d. *** *P* < 0.001, ** *P* < 0.01, * *P* < 0.05, n.s., not significant. (J) Mitochondrial morphology in a single body wall muscle cell in animals with indicated RNAi treatments. Arrows show the swollen mitochondrial structure. Scale bar, 5 μm. (K) Percentage of embryos with P*hsp-6*::GFP punctate patterns after indicated RNAi treatments. *n* > 70 per group. Data represent 3 biological replicates. Mean ± s.d. *** *P* < 0.001, ** *P* < 0.01, n.s., not significant. (L) Mitochondrial membrane potential (ΔΨm) in animals with indicated RNAi treatments. ΔΨm were indicated by TMRE. Data represent 3 biological replicates. (M) ROS levels in animals with indicated RNAi treatments. ROS levels were indicated by the sensor reporter P*rpl-17*::HyPer. Data represent 5 biological replicates.

Chromosome segregation errors cause genotoxic stress and lead to genome instability [60]. We explored whether agents causing DNA damage affect mitochondrial fission. Hydroxyurea (HU) and cisplatin stall DNA replication fork to interfere with genome integrity. UV-C radiation distorts DNA structure mainly by covalently linking adjacent pyrimidines, introducing DNA base lesions. ROS is a common threat to genome integrity. Potent oxidizers, like hydrogen peroxide (H_2_O_2_), generate ROS that causes DNA damage. Bleomycin and etoposide can cause DNA double-strand breaks (DSBs). We treated the animals with low doses of these chemicals to induce genome instability. All treatments caused a high frequency of aberrant karyotypes compared to the controls (Figs 7A-C and S6D left), indicating that these treatments disrupt genome integrity in somatic cells. We found that treatments with these DNA damage agents all caused mitochondrial elongation and hyperfusion in body wall muscle cells (Figs 7D-F, and S6A-S6E), suggesting that genome instability causes a mitochondrial fission defect. It is noted that these chemical treatments can also damage the mitochondrial genome. However, since the mitochondrial genome exists in multiple copies, it can withstand damage to some extent. Additionally, we used low doses of each chemical treatment. Notably, P*hsp-6*::GFP was not activated by these treatments, with the exception of UV-C (S6F-S6I Fig), suggesting that these chemical treatments did not significantly affect the mitochondrial genome integrity. Therefore, the mitochondrial fission defect observed following these chemical treatments is likely attributable to instability in the nuclear genome.

DNA damage is sensed and responded to by a family of phosphoinositide 3-kinase (PI3K)-related kinases (PIKKs) including ataxia-telangiectasia mutated (ATM) and Ataxia telangiectasia and Rad3-related (ATR) [61,62]. We found that RNAi of *pri-2*, *hcp-4*, and *knl-2* increased the expression of ATM-1 (the *C. elegans* orthologue of ATM). Although the ATR antibody did not work in our experiment, we found that RNAi of these genes resulted in the upregulation of phosphorylated CHK-1 (the *C. elegans* orthologue of CHK1), which is the major downstream substrate of ATR [63,64] (Figs 7G and S7A). This result suggests that inactivations of these identified genes, which cause improper chromosome segregation as shown before (Fig 6B and 6C), activate ATM and ATR. We then investigated whether ATM and/or ATR mediate the dysregulation of mitochondrial fission caused by improper chromosome segregation. RNAi of *atm-1* or *atl-1* (the *C. elegans* orthologue of *ATR*) did not activate P*hsp-6*::GFP in embryos, later-stage larvae or adults (Figs 7H and S7B). *atm-1* RNAi caused a mild decrease in mitochondrial membrane potential, as suggested by TMRE staining, whereas *atl-1* RNAi did not (S7D Fig). Neither *atm-1* nor *atl-1* RNAi affected the endogenous ROS level, as indicated by the P*rpl-17*::HyPer reporter (S7E Fig). Furthermore, RNAi of *atl-1* caused a mild mitochondrial elongation in body wall muscles, whereas *atm-1* RNAi did not affect mitochondrial morphology significantly (Fig 7I and 7J). Taken together, the inactivation of *atm-1/ATM* or *atl-1/ATR* has little effect on mitochondrial dynamics or mitochondrial function.

However, we found that RNAi of our newly identified mitochondrial fission genes including *pri-2*, *hcp-4*, and *knl-2*, in an ATL-1/ATR deficient background caused an even more severe mitochondrial fission defect in body wall muscles (Figs 7I, 7J, and S7C), showing that the elongated and hyperfused mitochondria were further tangled and retracted, while more blebbed or swollen mitochondrial structures appeared, suggesting a more severe mitochondrial fission defect (Fig 7J). Consistently, RNAi of *pri-2*, *hcp-4*, or *knl-2* in an ATL-1/ATR deficient background caused a further decrease in TOM20 peaks of mitochondrial plot profiles compared to their corresponding controls (Fig 7I) and an increase in mitochondrial lengths (S7C Fig). However, RNAi of *hcp-4* or *knl-2* in an ATM-1/ATM deficient background had little effect compared to their corresponding controls, though RNAi of *pri-2* in an ATM-1/ATM deficient background caused a more severe mitochondrial fission defect (Figs 7I, 7J, and S7C). Previously we indicated that the mitochondrial fission defect is closely associated with the embryonic punctate phenotype of the responsive reporters (Figs 2 and 3). Indeed, RNAi of *pri-2*, *hcp-4*, or *knl-2* in an ATL-1/ATR deficient background resulted in an even higher percentage of embryos exhibiting P*hsp-6*::GFP puncta (Fig 7K). Whereas RNAi of *pri-2* but not *hcp-4* or *knl-2* in an ATM-1/ATM deficient background increased the percentage of embryos exhibiting P*hsp-6*::GFP puncta compared to the corresponding control (Fig 7K). Quantitative RT-PCR analysis verified the efficient and generally consistent knockdown efficiency of the target genes in our RNAi experiments (S8A Fig). Moreover, we employed an *atm-1* loss-of-function mutant and the results were consistent with those obtained from the RNAi knockdown experiments (S8B-S8E Fig). Taken together, we suggest that ATL-1/ATR may play a protective role in mitochondrial fission defects triggered by chromosome segregation errors, while ATM-1/ATM may have a minor effect. Indeed, RNAi of *pri-2*, *hcp-4*, or *knl-2* in an ATL-1/ATR deficient background caused a further loss of mitochondrial membrane potential and a higher ROS level compared to the corresponding control, whereas inactivation of *atl-1* alone had little effect on either parameter (Figs 7L, 7M, and S7D and S7E). Importantly, overexpression of ATL-1/ATR alleviated the mitochondrial hyperfusion caused by *pri-2*, *hcp-4,* or *knl-2* inactivation in the body wall muscles (S7F-S7H Fig), suggesting that ATL-1/ATR potentially protects against mitochondrial fission defects caused by chromosome missegregation. ATM-1/ATM deficiency resulted in a further loss of mitochondrial membrane potential in response to *pri-2* or *hcp-4* RNAi but had little effect on mitochondrial membrane potential in *knl-2* RNAi. Additionally, there was minimal impact on ROS levels in the RNAi treatment of *pri-2*, *hcp-4,* or *knl-2* in the context of ATM-1/ATM inactivation (Fig 7L and 7M). These results suggest that ATL-1/ATR potentially alleviates mitochondrial fission defects triggered by chromosome missegregation, thereby maintaining mitochondrial function, while ATM-1/ATM may play a minor role.

## Discussion

The imbalance of mitochondrial fusion-fission is evident in various human diseases. Targeting mitochondrial dynamics is a promising therapeutic strategy. However, the regulatory genes and mechanisms for mitochondrial fusion or fission remain largely elusive. Changes in mitochondrial morphology are widely used for studying mitochondrial dynamics, which, however, rely heavily on high-resolution fluorescence images. A developmental phenotype in the *drp-1* loss-of-function mutant is linked to its fission activity [30]. In this study, we report that a mitochondrial fission defect causes a characteristic embryonic punctate pattern of stress reporter genes that respond to mitochondrial dysregulation. The embryonic punctate phenotype may be triggered by moderate to severe mitochondrial fission defects, as mild or modest fission dysfunction does not cause such a phenotype (S1D and S1E Fig). Both fission-associated phenotypes we reported previously [30] and in this study were readily adapted to a large-scale RNAi screen to identify new regulators of mitochondrial fission. It is expected to identify more genes involved in mitochondrial fission by expanding these screens to a whole genome scale.

mtUPR is activated by various mitochondrial dysfunctions. Studies have widely used P*hsp-6*::GFP for screening regulators of mitochondrial function and homeostasis [37,65–68]. However, our screen in this study differs from previous ones in several ways: 1) We demonstrate that the embryonic GFP punctate phenotype was observed not only with P*hsp-6*::GFP but also with another stress-responsive reporter, P*ugt-61*::GFP, suggesting the characteristic pattern is not specific to mtUPR. 2) Inactivation of most of the identified genes from our screen did not activate P*hsp-6*::GFP in the adult intestine (S3A and S4A Figs), a phenotype commonly used in prior screens [37,65–68]. This may explain why the fission regulator genes were challenging to identify in prior screens that relied on intestinal P*hsp-6*::GFP activation. 3) Thus, our screen identifies genes whose products mediate mitochondrial dynamics, as we demonstrated that the embryonic punctate pattern is tightly coupled to the mitochondrial fission defect. It has been reported that mild stress causes mitochondrial hyperfusion [69]. While our identified genes could affect mitochondrial dynamics in a stress-induced mitochondrial hyperfusion (SIMH) manner, SIMH typically leads to increased mitochondrial ATP production [69]. However, our data show that inactivation of the identified genes causes reduced mitochondrial membrane potential (S4D Fig), suggesting that SIMH is unlikely to be involved.

How does a mitochondrial fission defect give rise to the punctate phenotype of responsive reporters in embryos and larvae (Figs 1D, 1F, and S3B)? Mitochondrial fission plays a critical role in organelle quality control, primarily by facilitating the elimination of damaged mitochondria through mitophagy [70–72]. We found that the *drp-1* mutation especially induced P*hsp-6*::GFP at high levels in muscles and neurons (S3C and S3D Fig). These cell types have a particularly high demand for mitochondrial activity, making them more prone to generating damaged mitochondria even under normal growth conditions. While fusion functionality can compensate for minor mitochondrial impairments by sharing components among functional organelles, severe mitochondrial damage exceeds the capacity of fusion, necessitating fission-dependent mitophagy for organelle quality control. We assume that the mitochondrial fission defect disrupts mitophagy thus damaged mitochondria accumulate in muscles and neurons, which activates the responsive genes *in situ* to display the punctate phenotype in specifically damaged cells. Although we have excluded the involvement of PINK/Parkin-mediated mitophagy, it is possible that other fission-dependent mitophagy pathways or mitochondrial degradation systems function in this process. Furthermore, it is thought that there is a rejuvenation mechanism during mitochondrial inheritance, whereby daughter cells or gametes preferentially inherit healthy mitochondria over the damaged ones. Mitochondrial fragmentation and mitophagy may be required for such preferential mitochondrial inheritance and quality control [73]. Interestingly, we found that the fission defect-triggered punctate phenotype was passed on to the progeny embryos (Fig 1G), confirming that rejuvenation during mitochondrial inheritance is dependent on mitochondrial fission. It is of great interest to further investigate the underlying mechanism for such mitochondrial rejuvenation.

At each cell division, mitochondria as well as chromosomes are distributed to both daughter cells, so that the coupling of chromosome segregation regulation to mitochondrial fission regulation makes reasonable biological sense. But for chromosomes, each paternal and each maternal chromosome and not some aneuploid number must be segregated to each daughter cell, whereas for mitochondria, the distribution need not be so precise. But how the segregation of chromosomes is mechanistically coupled to the segregation of mitochondria needs comprehensive genetic study. Our data show that chromosome missegregation and genome instability dysregulate mitochondrial fission potentially independently of DRP-1, which is alleviated by ATL-1/ATR and possibly also ATM-1/ATM. This reveals a potential new pathway by which the nucleus regulates mitochondria, which may differ from the well-studied mechanisms by which cell cycle-related proteins affect mitochondrial dynamics by regulating DRP1 activity. ATR and ATM play essential roles in DNA damage response and repair to maintain nuclear genome integrity. Notably, our findings reveal a new function for ATR and perhaps also ATM in surveilling mitochondrial fission upon chromosome missegregation or genome instability. Such surveillance of mitochondrial fission quality might be considered part of ATR-mediated DNA damage responses. Chromosome segregation errors cause aneuploidy, a hallmark of cancer [74,75]. Our work reveals that aneuploidy is closely coupled to defective mitochondrial dynamics and function. Aneuploidy in cancer may also be coupled with aberrant mitochondrial fission and fusion in tumor cells.

## Materials and methods

### *C. elegans* strains and maintenance

Unless otherwise specified, all *C. elegans* strains used in this study were cultured on standard nematode growth medium (NGM) inoculated with *E. coli* OP50-1 (streptomycin resistant) bacteria at 20 °C. Gravid hermaphrodites were synchronized using the standard bleaching buffer, and the resulting embryos were incubated in M9 buffer overnight until they reached the L1 larval stage. The following *C. elegans* strains were used in this study: N2 Bristol: wild-type, CU6372: *drp-1(tm1108)IV*, PS6192: *syIs243*[P*myo-3*::TOM20::mRFP], SJ4100: *zcIs13*[P*hsp-6*::GFP]*V*, JV1: *jrIs1*[P*rpl-17*::HyPer + *unc-119*(+)]*III*, DA631: *eat-3(ad426)II*; *him-8(e1489)IV*, SJ4143: *zcIs17*[P*ges-1*::GFP(mit)], VC381: *atm-1(gk186)I*, JS71: *dpy-11(*e224*)* *air-1(*vw5*)V/eT1* *(III;V)*, EU630: *air-2(*or207*)I*, JS604: *dpy-17(*e164*)* *tlk-1(*tm2395*)III*/*hT2* [*bli-4(*e937*) let-?(*q782*) qIs48*] *(I;III)*;*knl-2(tm7587*)**, VC726: *hcp-4(*ok1057*)I*/*hT2* [*bli-4(*e937*) let-?(*q782*)* *qIs48*] *(I;III)*, LW905: *lmn-1(*tm1502*)I*/*hT2* [*bli-4(*e937*)* *let-?(*q782*)* *qIs48*] *(I;III)*, WIV1: *baxIs1*[P*ugt-61*::GFP] was generated by UV-integrated from BC11571: *sEx11571*[rCes P*ugt-61*::GFP + pCeh361] and outcrossed 6X, GR3065: translational *sur-5*::GFP from Gary Ruvkun laboratory stock, *drp-1(or1393)IV* was generated by out-crossing EU2706: *ruIs32*[P*pie-1*::GFP::H2B]*III*; *drp-1(or1393)IV* into N2, *drp-1(or1393)*; P*myo-3*::TOM20::mRFP was generated by crossing *drp-1(or1393)* into P*myo-3*::TOM20::mRFP, *drp-1(or1393)*; P*hsp-6*::GFP was generated by crossing *drp-1(or1393)* into P*hsp-6*::GFP, *drp-1(tm1108)*; P*hsp-6*::GFP was generated by crossing *drp-1(tm1108)* into P*hsp-6*::GFP. *air-1(vw5)*;P*myo-3*::TOM20::mRFP was generated by crossing *air-1(vw5)* into P*myo-3*::TOM20::mRFP, *air-2(or207)*;P*myo-3*::TOM20::mRFP was generated by crossing *air-2(or207)* into P*myo-3*::TOM20::mRFP, *tlk-1(tm2395)*;P*myo-3*::TOM20::mRFP was generated by crossing *tlk-1(tm2395)* into P*myo-3*::TOM20::mRFP, *knl-2(tm7587)*;P*myo-3*::TOM20::mRFP was generated by crossing *knl-2(tm7587)* into P*myo-3*::TOM20::mRFP, *hcp-4(ok1057)*;P*myo-3*::TOM20::mRFP was generated by crossing *hcp-4(ok1057)* into P*myo-3*::TOM20::mRFP, *lmn-1(tm1502)*;P*myo-3*::TOM20::mRFP was generated by crossing *lmn-1(tm1502)* into P*myo-3*::TOM20::mRFP. *atl-1* OE;P*myo-3*::TOM20::mRFP was generated by injecting *pNL43*[P*myo-3*::GFP::ATL-1::*tbb-2* 3'UTR+*unc119* (+)] plasmid into *syIs243*[P*myo-3*::TOM20::mRFP] gonadal cells. Strains were provided by the Caenorhabditis Genetics Center, which is funded by the NIH Office of Research Infrastructure Programs (P40 OD010440).

### RNA interference

Animals were synchronized to the L1 stage and subsequently transferred to RNAi plates that had been seeded with the designated *E. coli* HT115 (DE3) RNAi strain. For experiments assessing mitochondrial morphology, RNAi was performed on L4-stage animals for 24 hours. The animals were then transferred to new RNAi plates and allowed to lay eggs for 2 hours. The progeny continued to grow on the RNAi plates until reaching adulthood. Plates were maintained at 20 °C throughout the experiment. To culture the dsRNA-expressing bacteria, a single bacterial colony was picked and cultured overnight in an LB liquid medium supplemented with 50 mg/mL ampicillin (Sigma Aldrich) at 37 °C. The enriched bacterial solution was then pelleted, resuspended in M9 buffer, and seeded onto 60 mm NGM plates containing 50 mg/mL ampicillin and 0.4 mM IPTG. The plates were incubated overnight to allow IPTG to take effect. The animals were cultured on either the indicated RNAi bacteria or empty vector control bacteria for 2–3 days. In the case of the dual RNAi experiment, equal volumes of the two RNAi bacteria were combined for use. It should be noted that the knockdown efficiency can vary between genes.

### TMRE, NAO and DAPI staining

For adult experiments, synchronized L1 animals were transferred to indicated RNAi plates and incubated at 20 °C for 2 days. Once the animals reached the late L4 stage, they were exposed to 100 μL of 2.5 μM TMRE (Tetramethylrhodamine, ethyl ester; Life Technologies) that covered the bacterial lawn. The plates were air-dried and incubated overnight at 20 °C to allow fluorescent labeling. Subsequently, the animals were transferred to new RNAi plates without TMRE and cultured for an additional 6–8 hours to remove any excess fluorescent dye. The images were captured using a Zeiss AxioZoom V16 microscope with consistent exposure time and magnification settings.

For embryonic experiments, synchronized L1 animals were transferred to indicated RNAi plates and incubated at 20 °C until they reached the first day of adulthood. Approximately 50 adult animals were then transferred to a polylysine-coated slide using 10 μL of M9 buffer. To release the embryos, the animals were cross-cut with an injection needle. Subsequently, 10 μL of a 5 μM solution of TMRE or 10 μL of a 100 ng/mL solution of Antifade Mounting Medium with DAPI (Beyotime) was added dropwise to the slide. The slides were then incubated at room temperature for 1 hour. For NAO staining, adult animals were harvested and fixed with 5% paraformaldehyde at room temperature for 30 min. After fixation, the slides were washed with M9 solution and incubated in ice-cold methanol at -20 °C for 20 min to facilitate penetration. Following another wash with M9, 5 μM NAO (Biorigin, BN14002) was added to the slides and incubated at room temperature for 30 min before imaging. Images were captured using a Zeiss AxioZoom M2 microscope.

### Measurement of reactive oxygen species (ROS)

P*rpl-17*::HyPer transgenic animals were synchronized to the L1 stage and subsequently transferred to the indicated RNAi plates. They were cultured until reaching the first day of adulthood. To anesthetize the animals, 20 μL of a 100 mM levamisole solution was added. The images were acquired using a Zeiss AxioZoom V16 microscope, with consistent exposure time and magnification settings.

### Mitochondrial morphology and length

Mitochondrial morphology was assessed using P*myo-3*::TOM20::mRFP or P*ges-1*::GFP(mit) as markers, and adult animals were immobilized on a 2% agarose pad for microscopic imaging. 8–10 body wall muscle cells in the middle of the worm body were examined for mitochondrial morphology, with a total of 10–15 animals chosen for each treatment. Mitochondria were categorized as “tubular morphology” if tubular structures dominated throughout a single muscle cell, “elongated morphology” if elongated structures predominated, and “fragmented morphology” if mitochondria appeared fragmented. Fluorescence images of mitochondrial morphology were captured using a Zeiss AxioZoom M2 microscope and a Leica TCS SP8 microscope.

Mitochondrial length was quantified using ImageJ software. Images were pre-processed by applying the following filters: Unsharp mask, Enhance local contrast, and Median. Afterward, the images were converted into binary form to generate a morphological skeleton, which allowed for the calculation of the “branch length” as a measure of mitochondrial length. Statistical analysis was performed using the Mann-Whitney U test with Prism 8 software.

### Induction of GFP reporters in animals

Animals were synchronized to the L1 stage and subsequently transferred to NGM plates with the indicated treatment. They were allowed to grow into adults. Dropped 5 μL of 100 mM levamisole onto a new standard NGM plate without bacteria. Afterward, several adult animals were transferred to the levamisole droplet for anesthetization and image capture. All images were acquired using a Zeiss AxioZoom V16 microscope at a fixed exposure time and magnification. To evaluate the fluorescence intensity of GFP, the entire body of the animal was outlined, and the fluorescence intensity was quantified using Zeiss software. Each experiment analyzed 10–20 animals per group, with at least three biological replicates.

### Induction of GFP reporters in embryos

Synchronized L1 animals were cultured on standard NGM plates until they reached the L4 stage. Subsequently, they were transferred to the designated RNAi plates. The animals were cultured on these RNAi plates for an additional 2 days at 20 °C to ensure sufficient embryo production. The embryonic fluorescent patterns were observed and captured using a ZEISS AxioZoom V16 microscope with the same magnification and exposure time settings. Data were collected from a minimum of 70 embryos per group per replicate, with at least three independent biological replicates.

### Hydroxyurea treatment

Hydroxyurea was dissolved in DMSO to prepare a solution with a concentration of 100 mM and then added to a standard NGM plate coated with bacteria to a final concentration of 1 mM. Synchronized L1 animals were cultured on the NGM plate containing 1 mM hydroxyurea for 60 hours until reaching adulthood.

### UV-C treatment

Early L1 larvae, growing on NGM agar plates coated with bacteria, were placed in a Spectrolinker UV Crosslinkers XL-1500 for UV-C irradiation. The UV irradiation was conducted using a UV wavelength of 254 nm and a UV irradiation dose of 2 x 10^5^ J/m^2^. After irradiation, plates were immediately returned to the incubator, and the animals were grown to adulthood.

### Hydrogen peroxide treatment

To the standard NGM agar plates, 50 μL of a 3% hydrogen peroxide solution was added and spread evenly to cover the entire bacterial lawn. The plates were then air-dried. Synchronized L1 animals were placed onto the plates containing hydrogen peroxide and grown to adulthood.

### Bleomycin, etoposide, and cisplatin treatments

Synchronized animals were grown on NGM plates to adulthood. The animals were then collected, washed with M9 solution, and incubated in the chemical solutions with rotation for 3 hours at room temperature. The chemical concentrations used were 50 μM bleomycin (Sangon, A418304), 100 μM etoposide (Sangon, A430901), and 50 μg/mL cisplatin (Gersion, BQS132930).

### Microscopy and observation of the morphology of the intestinal nucleus

Gravid *sur-5*::GFP animals were bleached and synchronized, and the resulting early L1 larvae were placed on NGM plates with the indicated treatment and cultured to adulthood. Several adult *sur-5*::GFP animals were mounted on a 2% agarose pad and anesthetized using 1.5 M sodium azide for microscopic observation. All images were captured using a Zeiss AxioZoom M2 microscope, with a fixed exposure time and magnification. Each experiment analyzed 10–20 animals per group and was performed in at least three biological replicates.

### Western blotting and antibodies

Animals were synchronized to the L1 stage and cultured on plates with the indicated treatment at 20 °C until adulthood. Afterward, the animals were harvested and washed with ddH_2_O 2–3 times. They were then resuspended to a final concentration of 1 × SDS Loading Buffer containing 5% β-mercaptoethanol and heated at 95 °C for 10 min. The lysate was loaded onto a Bis-Tris protein gel, with a 4% stacking gel and an 8% or 10% separating gel, and transferred to a nitrocellulose membrane (Amersham). The membrane was blocked with TBST containing 5% BSA (Beyotim) and probed with the designated primary and secondary antibodies. The primary antibodies used in this study included anti-ATM (ABclonal A5908, 1:1000), Phospho-Chk1(S317) antibody (Bethyl Laboratories A300-163A, 1:1000), anti-Actin (abcam ab179467, 1:2000). The secondary antibody used was horseradish peroxidase-labeled goat anti-rabbit IgG (H + L) (Beyotim A0208, 1:2000). Western blots were imaged using the Amersham Imager 600 and ImageQuant LAS4000 mini machine and further quantified using ImageJ software.

### Statistical analysis

At least three biological replicates were performed for each quantitative experiment, and samples were randomly selected. The sample size was not predetermined. Statistical tests were chosen based on the data distribution and variance characteristics, considering the appropriate underlying assumptions. Mann-Whitney test was used for analyzing data related to mitochondrial morphology experiments, while two-tailed unpaired t-tests were used for other experiments. Statistical analysis was performed using GraphPad Prism 8 and WPS Office 11. Information regarding the specific statistical tests, p-values, and the number of significant digits is provided in the corresponding figure captions and legends.

### Experimental study design

Some experiments and statistical analyses were conducted in a blinded manner to minimize potential bias. Specifically, the researchers performing the experiments or analyzing the data or images were unaware of the treatment group assignments.

## Supporting information

S1 Fig DRP-1 deficiency causes a characteristic punctate pattern of the responsive reporters in *C. elegans* embryos. (*A*) Mitochondrial morphology in a single body wall muscle cell in animals with indicated RNAi treatments. Mitochondrial lengths in body wall muscles in animals with indicated RNAi treatments. Each data point represents the mitochondrial length measured from an animal body wall muscle cell. *n* = 41–50 per group. Median with 95% C. I. Mann-Whitney test. ****P < 0.0001, **P < 0.01. Scale bar, 5 μm. (*B*) P*hsp-6*::GFP expression in animals with indicated RNAi treatments. Scar bar, 0.2 mm. (*C*) P*hsp-6*::GFP activation patterns in animals with indicated RNAi treatments. (*D*) Mitochondrial morphology in a single body wall muscle cell in indicated animals. Mitochondrial lengths in body wall muscles in animals with indicated RNAi treatments. Each data point represents the mitochondrial length measured from an animal body wall muscle cell. *n* = 41–60 per group. Median with 95% C. I. Mann-Whitney test. ****P < 0.0001, **P < 0.01. TOM20 peak number for the plot profiles of mitochondrial morphology in indicated animals. Each data point represents the median TOM20 peak number from the plot profile of mitochondrial morphology in a muscle cell. *n* = 10 per group. Mean ± s.d. *P < 0.05. Scale bar, 5 μm. (*E*) P*hsp-6*::GFP expression in embryos in indicated animals.
(TIF)

S2 Fig Representative plot profiles of mitochondrial morphological images from indicated animals. The yellow lines mark the different cross-sections of the images. The number indicates the TOM20 peak.
(TIF)

S3 Fig *drp-1* mutation activates P*hsp-6*::GFP especially in muscles and neurons. (*A*) P*hsp-6*::GFP expression in animals with indicated RNAi treatments. Scar bar, 0.2 mm. (*B*) P*hsp-6*::GFP expression in *drp-1(tm1108)* larvae. (*C*) P*hsp-6*::GFP activation in body wall muscles in *drp-1(tm1108)*. (*D*) P*hsp-6*::GFP activation in various neural structures and cells in *drp-1(tm1108)*. (*E*) P*hsp-6*::GFP expression in embryos in indicated animals. (*F*) Mitochondrial morphology in a single body wall muscle cell in animals with indicated RNAi treatments. Scale bar, 5 μm.
(TIF)

S4 Fig Inactivation of the identified genes affects mitochondrial function. (*A*) P*hsp-6*::GFP expression in animals with indicated RNAi treatments. Scale bar, 0.2 mm. (*B*) Mitochondrial morphology in a single body wall muscle cell (left), TOM20 peak number for the plot profiles of mitochondrial morphology (middle, Mean ± s.d.), and mitochondrial lengths body wall muscles (right, Median with 95% C. I. Mann-Whitney test) in indicated animals. ****P < 0.0001, ***P < 0.001, **P < 0.01. Scale bar, 5 μm. (*C*) Mitochondrial morphology in animal intestine after indicated RNAi treatments. Zoomed-in fluorescence images (left bottom). Scale bar, 15 μm. (*D*) Mitochondrial membrane potential (ΔΨm) in animals with indicated RNAi treatments. ΔΨm were indicated by Tetramethylrhodamine ethyl ester (TMRE). Each data point represents the fluorescence intensity measured from a single animal. *n* = 5 per group. (*E*) ROS levels in animals with indicated RNAi treatments. ROS levels were indicated by the sensor reporter P*rpl-17*::HyPer. Each data point represents the fluorescence intensity measured from a single animal. *n* = 5 per group. (*F*) Mitochondrial distribution in early embryos with indicated RNAi treatments. Mitochondria were indicated by NAO. Scale bar, 10 μm. (*G*) Percentage of embryos with P*hsp-6*::GFP punctate patterns in wild-type (WT) and *drp-1 loss-of-function (lf)* animals after indicated RNAi treatments. *n* > 150 per group. Data represent 3 biological replicates. Mean ± s.d. Data related to Fig 5B.
(TIF)

S5 Fig Inactivation of the identified genes causes improper chromosome segregation. (*A*) P*xol-1*::GFP induction in animals with indicated RNAi treatments. (*B*) Penetrance of embryonic lethality in animals with indicated RNAi treatments. *n* = 90–110 per group.
(TIF)

S6 Fig Genome instability causes a mitochondrial fission defect. (*A*-*C*) Mitochondrial lengths in body wall muscles in animals with indicated treatments. Each data point represents the mitochondrial length measured from an animal body wall muscle cell. *n* = 30–40 per group. Median with 95% C. I. Mann-Whitney test. **P < 0.01, *P < 0.05. (*D*) (Left) Postembryonic cell divisions in animal intestines shown by *sur-5*::GFP with indicated treatments. Arrows show abnormal karyotypes. (Right) Mitochondrial morphology in a single body wall muscle cell in animals with indicated treatments. Scale bar, 5 μm. (*E*) TOM20 peak number for the plot profiles of mitochondrial morphology (left, Mean ± s.d.), and mitochondrial lengths body wall muscles (right, Median with 95% C. I. Mann-Whitney test) in animals with indicated treatments. ****P < 0.0001, ***P < 0.001. (*F*-*I*) P*hsp-6*::GFP expression in animals with indicated treatments. Each data point represents the fluorescence intensity measured from a single animal. *n* > 15 per group.
(TIF)

S7 Fig ATR alleviates mitochondrial fission defects triggered by chromosome missegregation. (*A*) Relative protein expression levels. Data represent 5 biological replicates. Mean ± s.d. Mann-Whitney test. **P < 0.01. (*B*) P*hsp-6*::GFP expression in animals with indicated RNAi treatments. Scale bar, 0.2 mm. (*C*) Mitochondrial lengths in body wall muscles in animals with indicated RNAi treatments. Each data point represents the mitochondrial length measured from an animal body wall muscle cell. *n* = 10–20 per group. Median with 95% C. I. Mann-Whitney test. *P < 0.05, n.s., not significant. (*D*) Mitochondrial membrane potential (ΔΨm) in animals with indicated RNAi treatments. ΔΨm were indicated by TMRE. Each data point represents the fluorescence intensity measured from a single animal. *n* = 5 per group. Mean ± s.d. *P < 0.05, n.s., not significant. (*E*) ROS levels in animals with indicated RNAi treatments. ROS levels were indicated by the sensor reporter P*rpl-17*::HyPer. Each data point represents the fluorescence intensity measured from a single animal. *n* = 5 per group. Mean ± s.d. n.s., not significant. (*F*) Mitochondrial morphology in a single body wall muscle cell in animals with or without ATL-1 overexpression (*atl-1* OE) after indicated RNAi treatments. Scale bar, 5 μm. (*G*) TOM20 peak number for the plot profiles of mitochondrial morphology in animals with or without ATL-1 overexpression after indicated RNAi treatments. Each data point represents the median TOM20 peak number from the plot profile of mitochondrial morphology in a muscle cell. *n* = 10 per group. Mean ± s.d. ****P < 0.0001, *P < 0.05, n.s., not significant. (*H*) Mitochondrial lengths in body wall muscles in animals with or without ATL-1 overexpression after indicated RNAi treatments. Each data point represents the mitochondrial length measured from an animal body wall muscle cell. *n* = 20–30 per group. Median with 95% C. I. Mann-Whitney test. **P < 0.01, *P < 0.05, n.s., not significant.
(TIF)

S8 Fig ATM-1/ATM has a minor effect on alleviating mitochondrial fission defects caused by chromosome missegregation. (*A*) Evaluation of RNAi efficiency by quantitative RT-PCR. Data represent 3 biological replicates. Mean ± s.d. ****P < 0.0001, ***P < 0.001. (*B*) Mitochondrial morphology in a single body wall muscle cell in wild-type (WT) and *atm-1(gk186)* animals after indicated RNAi treatments. Scale bar, 5 μm. (*C*) Percentage of embryos with P*hsp-6*::GFP punctate patterns in wild-type (+) and *atm-1 loss-of-function (-)* animals after indicated RNAi treatments. *n* > 150 per group. Data represents 3 biological replicates. Mean ± s.d. **P < 0.01, *P < 0.05, n.s., not significant. (*D*) TOM20 peak number for the plot profiles of mitochondrial morphology in wild-type (+) and *atm-1 loss-of-function (-)* animals with indicated RNAi treatments. Each data point represents the median TOM20 peak number from the plot profile of mitochondrial morphology in a muscle cell. *n* = 10 per group. Mean ± s.d. *P < 0.05, n.s., not significant. (*E*) Mitochondrial lengths in body wall muscles in wild-type (+) and *atm-1 loss-of-function (-)* animals with indicated RNAi treatments. Each data point represents the mitochondrial length measured from an animal body wall muscle cell. *n* = 20–30 per group. Median with 95% C. I. Mann-Whitney test. *P < 0.05, n.s., not significant.
(TIF)

S1 Data Gene list of the kinase library. (XLSX)

S2 Data Gene list of 3,300 random genes used in the RNAi screen. (XLSX)

S3 Data Primary data underlying graphs in figures. (XLSX)
