# Supplementary figures and images for: Mitochondrial fission surveillance is coupled to Caenorhabditis elegans DNA and chromosome segregation integrity
Source: PLoS Genet. 2025 Apr 25;21(4):e1011678. doi: 10.1371/journal.pgen.1011678 (PMC12064022; doi:10.1371/journal.pgen.1011678)

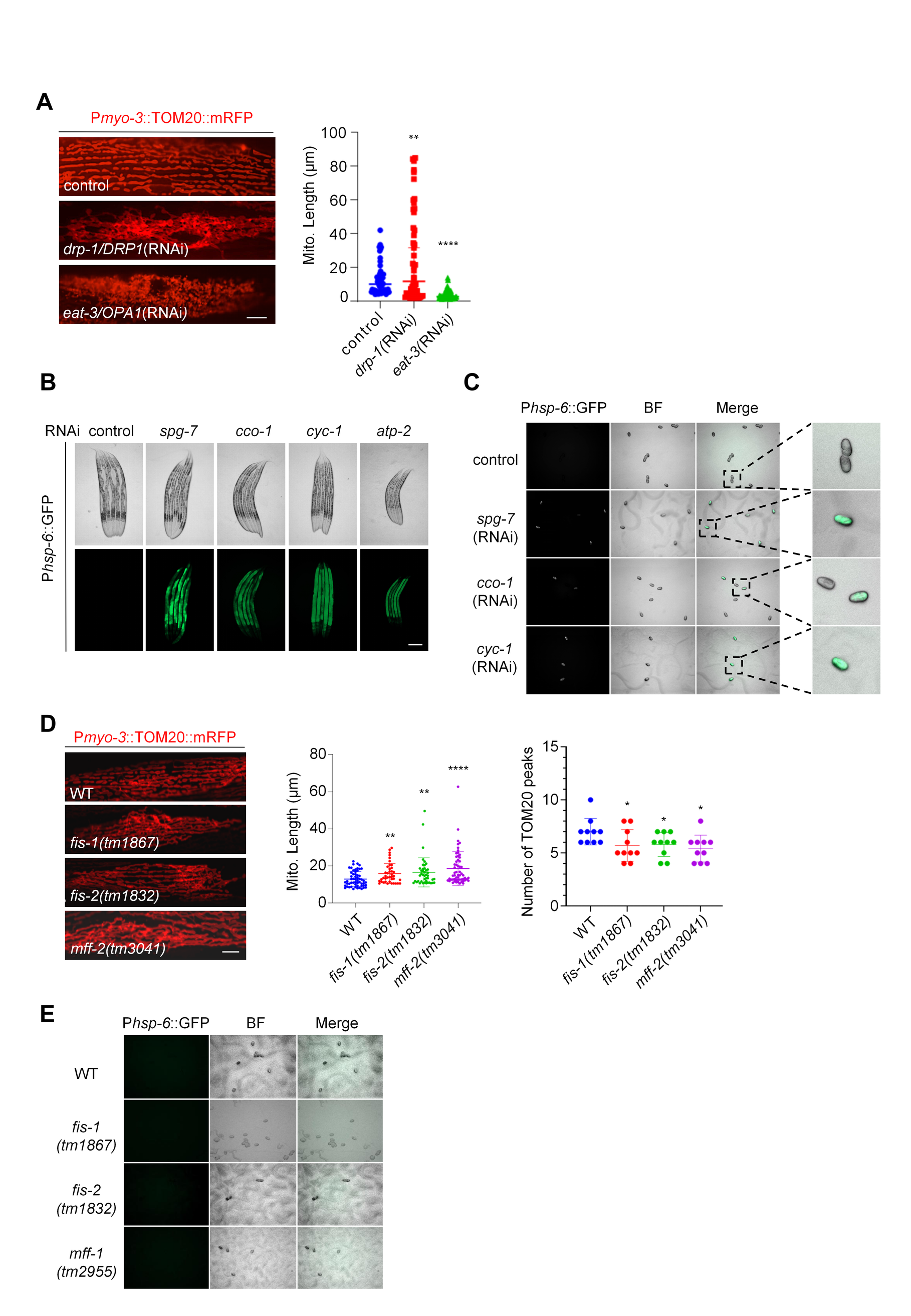

Supplement: S1 Fig — (A) Mitochondrial morphology in a single body wall muscle cell in animals with indicated RNAi treatments. Mitochondrial lengths in body wall muscles in animals with indicated RNAi treatments. Each data point represents the mitochondrial length measured from an animal body wall muscle cell. n = 41–50 per group. Median with 95% C. I. Mann-Whitney test. ****P < 0.0001, **P < 0.01. Scale bar, 5 μm. (B) Phsp-6::GFP expression in animals with indicated RNAi treatments. Scar bar, 0.2 mm. (C) Phsp-6::GFP activation patterns in animals with indicated RNAi treatments. (D) Mitochondrial morphology in a single body wall muscle cell in indicated animals. Mitochondrial lengths in body wall muscles in animals with indicated RNAi treatments. Each data point represents the mitochondrial length measured from an animal body wall muscle cell. n = 41–60 per group. Median with 95% C. I. Mann-Whitney test. ****P < 0.0001, **P < 0.01. TOM20 peak number for the plot profiles of mitochondrial morphology in indicated animals. Each data point represents the median TOM20 peak number from the plot profile of mitochondrial morphology in a muscle cell. n = 10 per group. Mean ± s.d. *P < 0.05. Scale bar, 5 μm. (E) Phsp-6::GFP expression in embryos in indicated animals. (TIF) [file pgen.1011678.s001.tif]

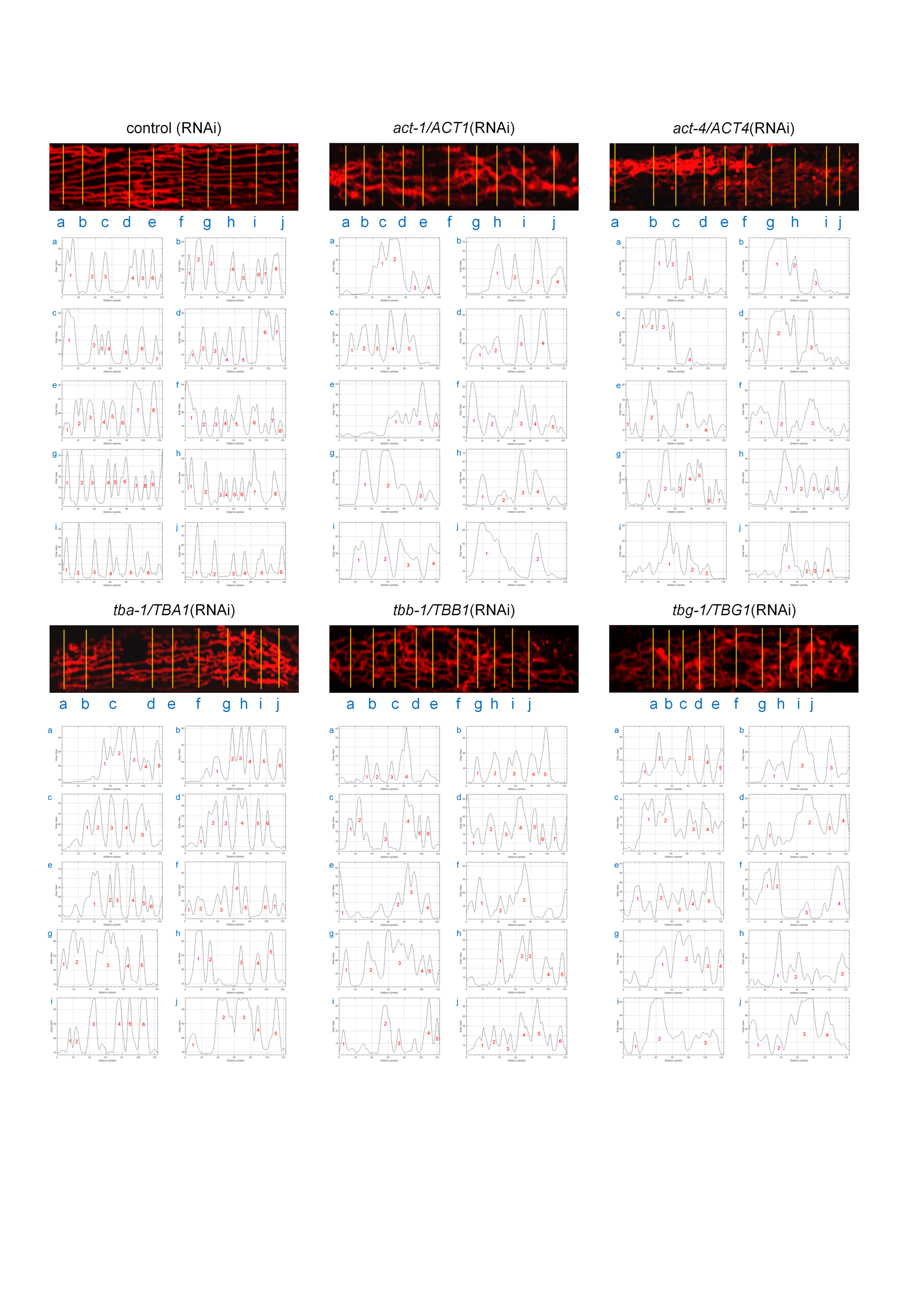

Supplement: S2 Fig — The yellow lines mark the different cross-sections of the images. The number indicates the TOM20 peak. (TIF) [file pgen.1011678.s002.tif]

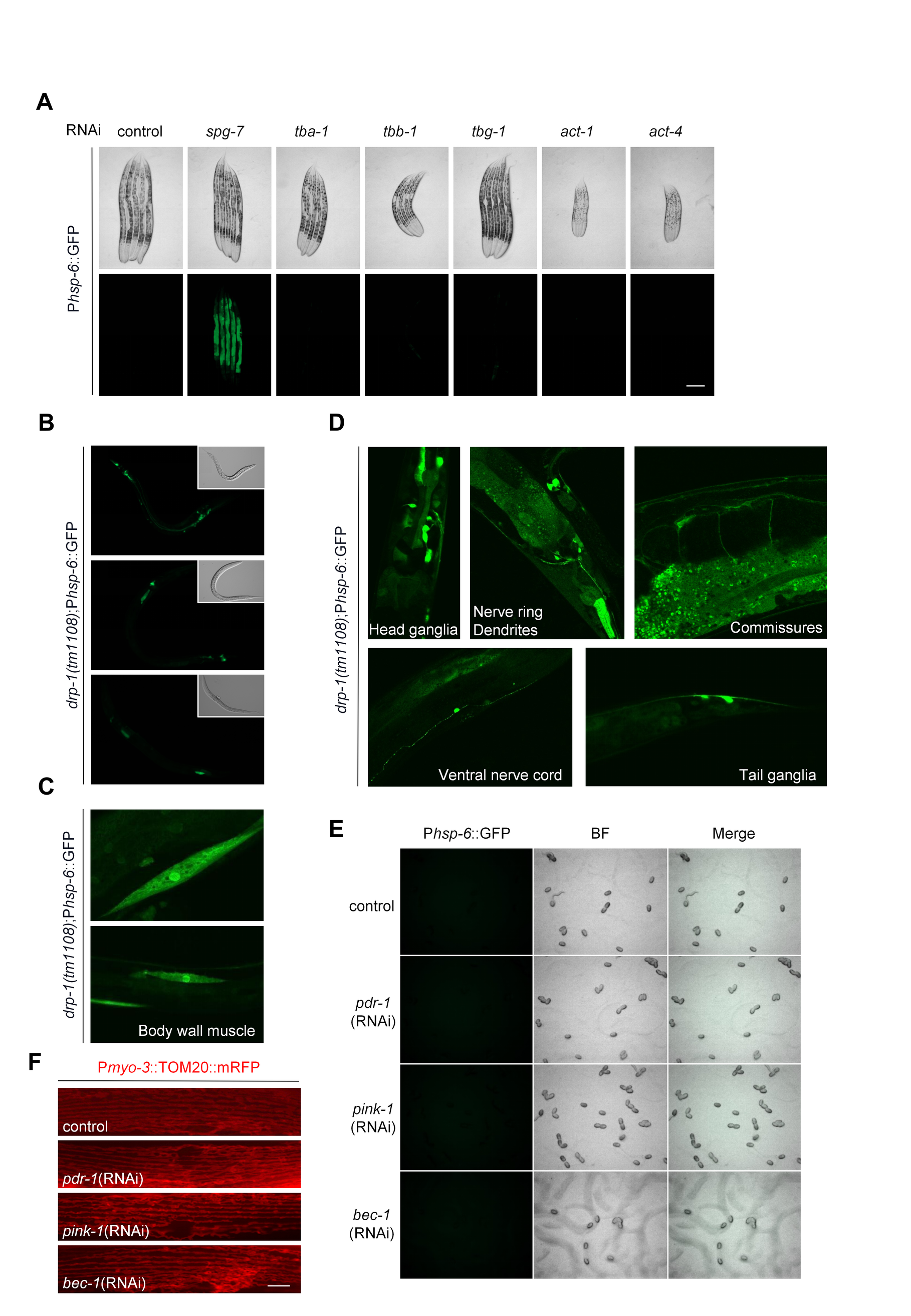

Supplement: S3 Fig — (A) Phsp-6::GFP expression in animals with indicated RNAi treatments. Scar bar, 0.2 mm. (B) Phsp-6::GFP expression in drp-1(tm1108) larvae. (C) Phsp-6::GFP activation in body wall muscles in drp-1(tm1108). (D) Phsp-6::GFP activation in various neural structures and cells in drp-1(tm1108). (E) Phsp-6::GFP expression in embryos in indicated animals. (F) Mitochondrial morphology in a single body wall muscle cell in animals with indicated RNAi treatments. Scale bar, 5 μm. (TIF) [file pgen.1011678.s003.tif]

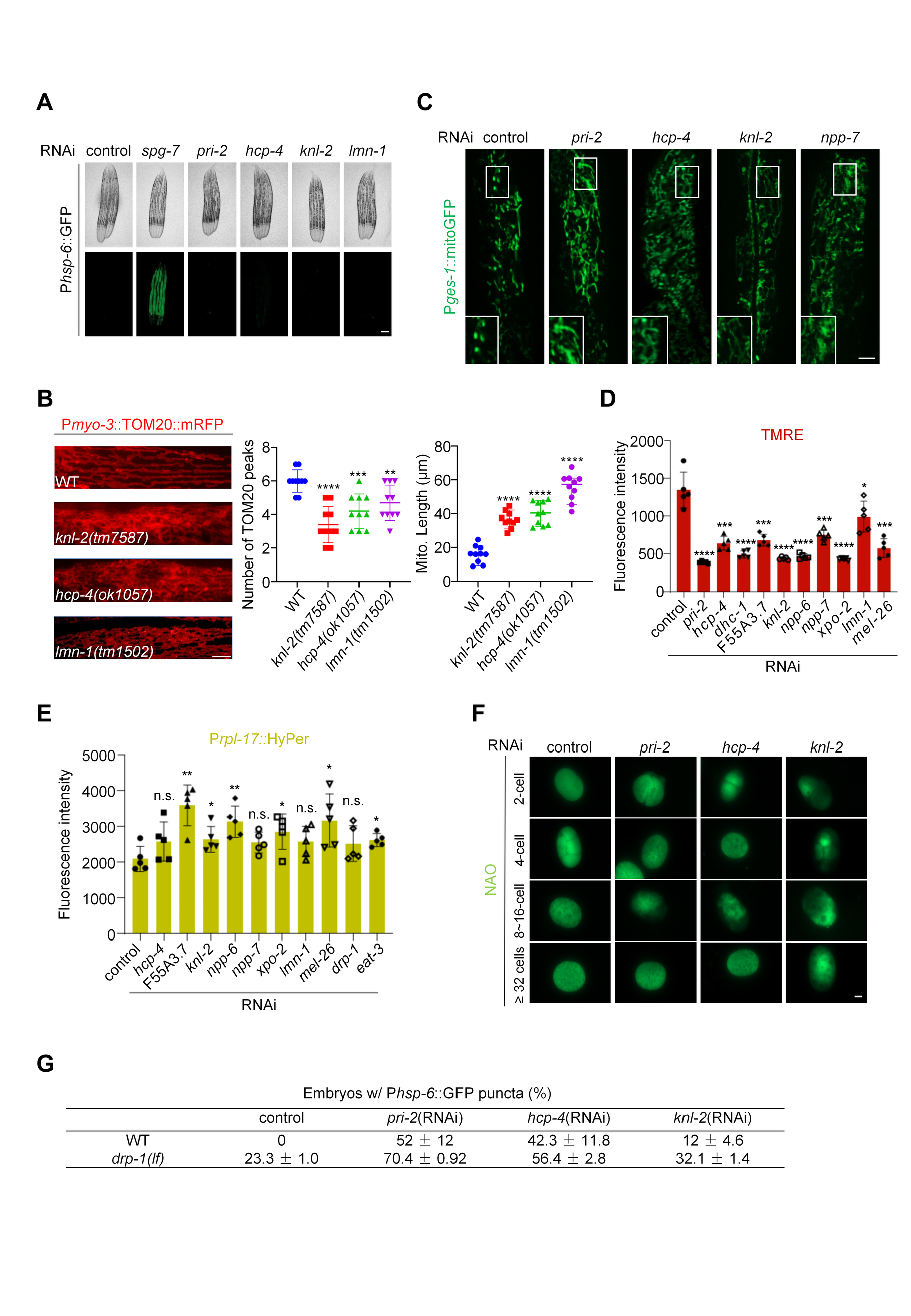

Supplement: S4 Fig — (A) Phsp-6::GFP expression in animals with indicated RNAi treatments. Scale bar, 0.2 mm. (B) Mitochondrial morphology in a single body wall muscle cell (left), TOM20 peak number for the plot profiles of mitochondrial morphology (middle, Mean ± s.d.), and mitochondrial lengths body wall muscles (right, Median with 95% C. I. Mann-Whitney test) in indicated animals. ****P < 0.0001, ***P < 0.001, **P < 0.01. Scale bar, 5 μm. (C) Mitochondrial morphology in animal intestine after indicated RNAi treatments. Zoomed-in fluorescence images (left bottom). Scale bar, 15 μm. (D) Mitochondrial membrane potential (ΔΨm) in animals with indicated RNAi treatments. ΔΨm were indicated by Tetramethylrhodamine ethyl ester (TMRE). Each data point represents the fluorescence intensity measured from a single animal. n = 5 per group. (E) ROS levels in animals with indicated RNAi treatments. ROS levels were indicated by the sensor reporter Prpl-17::HyPer. Each data point represents the fluorescence intensity measured from a single animal. n = 5 per group. (F) Mitochondrial distribution in early embryos with indicated RNAi treatments. Mitochondria were indicated by NAO. Scale bar, 10 μm. (G) Percentage of embryos with Phsp-6::GFP punctate patterns in wild-type (WT) and drp-1 loss-of-function (lf) animals after indicated RNAi treatments. n > 150 per group. Data represent 3 biological replicates. Mean ± s.d. Data related to Fig 5B. (TIF) [file pgen.1011678.s004.tif]

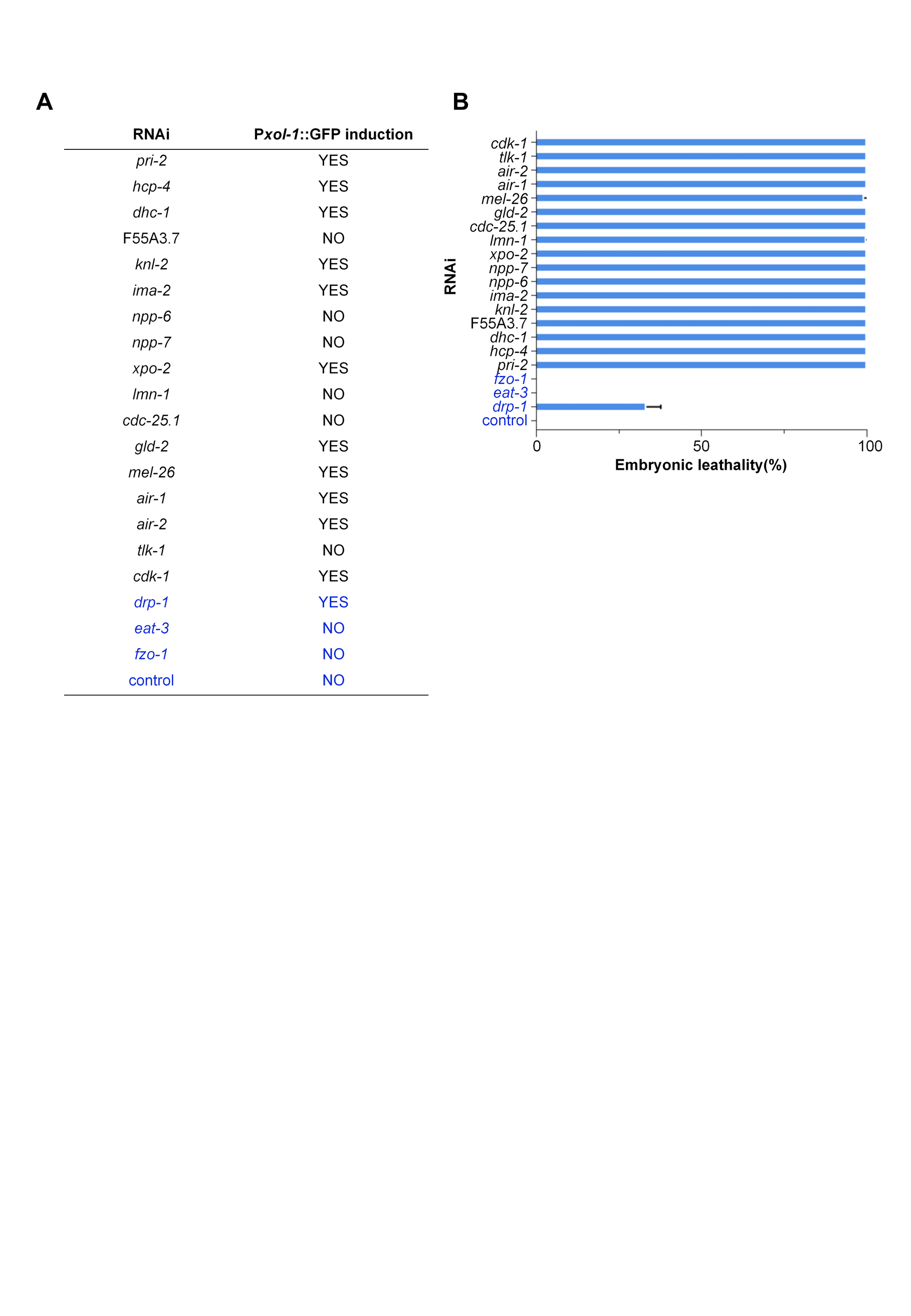

Supplement: S5 Fig — (A) Pxol-1::GFP induction in animals with indicated RNAi treatments. (B) Penetrance of embryonic lethality in animals with indicated RNAi treatments. n = 90–110 per group. (TIF) [file pgen.1011678.s005.tif]

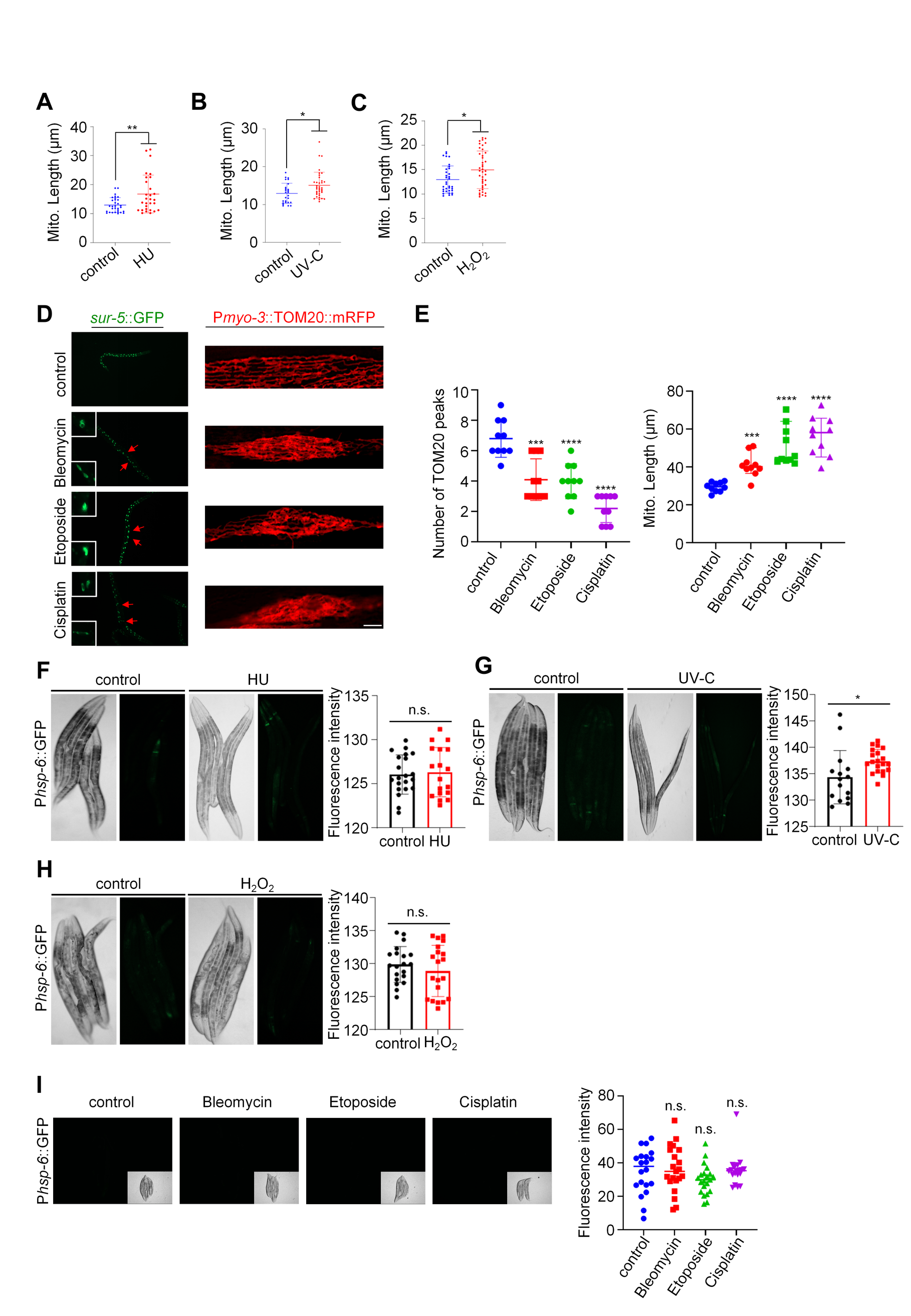

Supplement: S6 Fig — (A-C) Mitochondrial lengths in body wall muscles in animals with indicated treatments. Each data point represents the mitochondrial length measured from an animal body wall muscle cell. n = 30–40 per group. Median with 95% C. I. Mann-Whitney test. **P < 0.01, *P < 0.05. (D) (Left) Postembryonic cell divisions in animal intestines shown by sur-5::GFP with indicated treatments. Arrows show abnormal karyotypes. (Right) Mitochondrial morphology in a single body wall muscle cell in animals with indicated treatments. Scale bar, 5 μm. (E) TOM20 peak number for the plot profiles of mitochondrial morphology (left, Mean ± s.d.), and mitochondrial lengths body wall muscles (right, Median with 95% C. I. Mann-Whitney test) in animals with indicated treatments. ****P < 0.0001, ***P < 0.001. (F-I) Phsp-6::GFP expression in animals with indicated treatments. Each data point represents the fluorescence intensity measured from a single animal. n > 15 per group. (TIF) [file pgen.1011678.s006.tif]

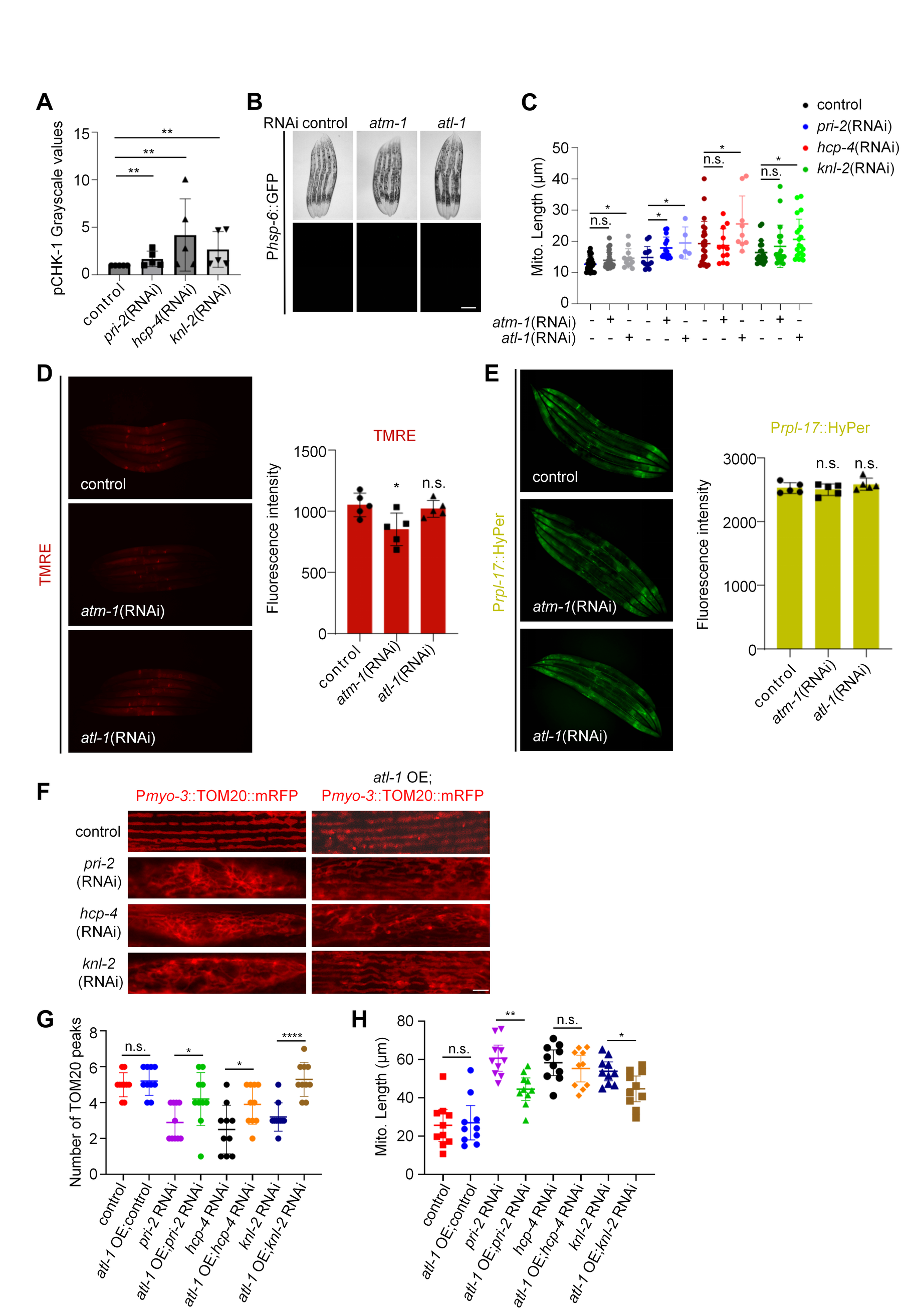

Supplement: S7 Fig — (A) Relative protein expression levels. Data represent 5 biological replicates. Mean ± s.d. Mann-Whitney test. **P < 0.01. (B) Phsp-6::GFP expression in animals with indicated RNAi treatments. Scale bar, 0.2 mm. (C) Mitochondrial lengths in body wall muscles in animals with indicated RNAi treatments. Each data point represents the mitochondrial length measured from an animal body wall muscle cell. n = 10–20 per group. Median with 95% C. I. Mann-Whitney test. *P < 0.05, n.s., not significant. (D) Mitochondrial membrane potential (ΔΨm) in animals with indicated RNAi treatments. ΔΨm were indicated by TMRE. Each data point represents the fluorescence intensity measured from a single animal. n = 5 per group. Mean ± s.d. *P < 0.05, n.s., not significant. (E) ROS levels in animals with indicated RNAi treatments. ROS levels were indicated by the sensor reporter Prpl-17::HyPer. Each data point represents the fluorescence intensity measured from a single animal. n = 5 per group. Mean ± s.d. n.s., not significant. (F) Mitochondrial morphology in a single body wall muscle cell in animals with or without ATL-1 overexpression (atl-1 OE) after indicated RNAi treatments. Scale bar, 5 μm. (G) TOM20 peak number for the plot profiles of mitochondrial morphology in animals with or without ATL-1 overexpression after indicated RNAi treatments. Each data point represents the median TOM20 peak number from the plot profile of mitochondrial morphology in a muscle cell. n = 10 per group. Mean ± s.d. ****P < 0.0001, *P < 0.05, n.s., not significant. (H) Mitochondrial lengths in body wall muscles in animals with or without ATL-1 overexpression after indicated RNAi treatments. Each data point represents the mitochondrial length measured from an animal body wall muscle cell. n = 20–30 per group. Median with 95% C. I. Mann-Whitney test. **P < 0.01, *P < 0.05, n.s., not significant. (TIF) [file pgen.1011678.s007.tif]

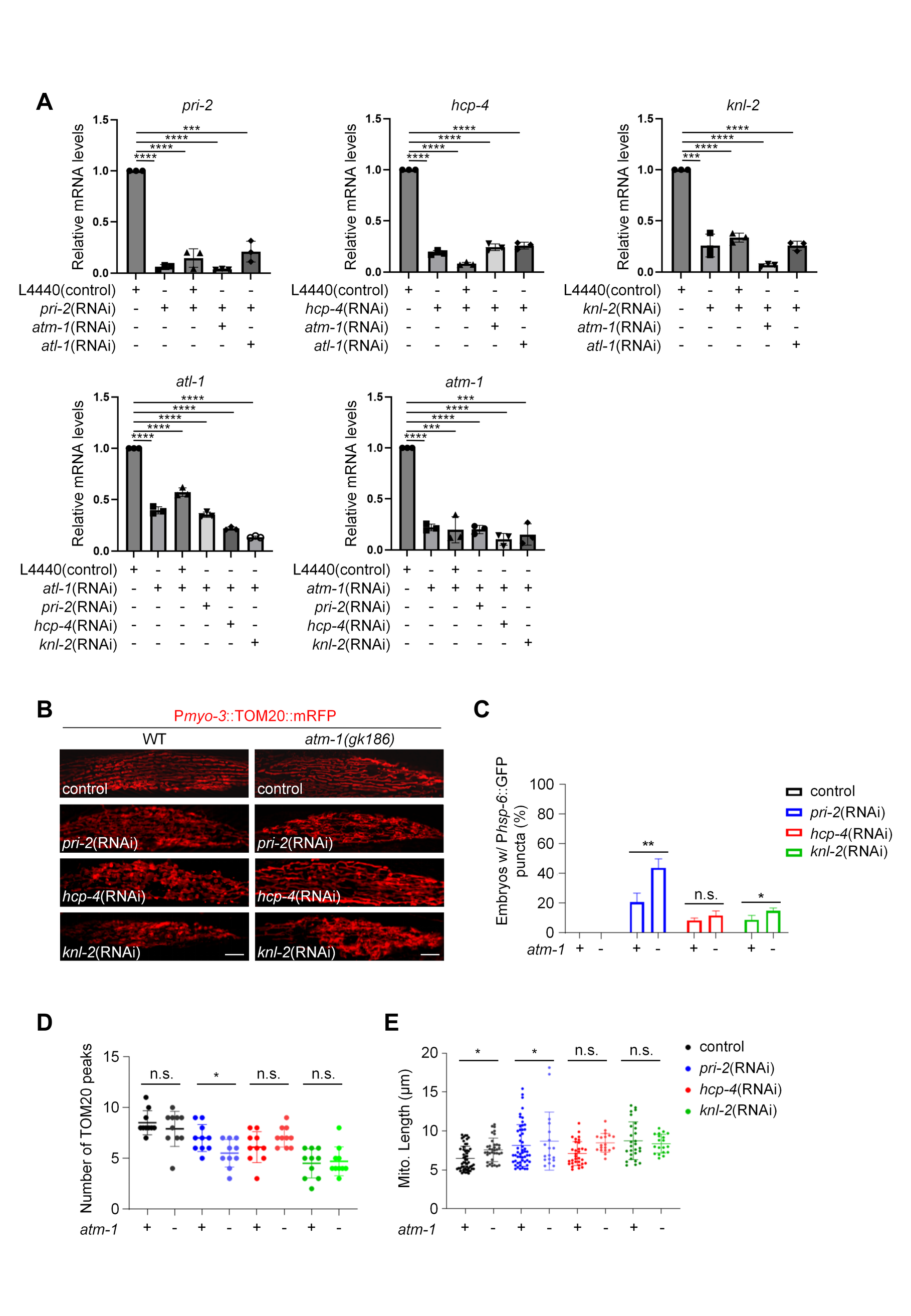

Supplement: S8 Fig — (A) Evaluation of RNAi efficiency by quantitative RT-PCR. Data represent 3 biological replicates. Mean ± s.d. ****P < 0.0001, ***P < 0.001. (B) Mitochondrial morphology in a single body wall muscle cell in wild-type (WT) and atm-1(gk186) animals after indicated RNAi treatments. Scale bar, 5 μm. (C) Percentage of embryos with Phsp-6::GFP punctate patterns in wild-type (+) and atm-1 loss-of-function (-) animals after indicated RNAi treatments. n > 150 per group. Data represents 3 biological replicates. Mean ± s.d. **P < 0.01, *P < 0.05, n.s., not significant. (D) TOM20 peak number for the plot profiles of mitochondrial morphology in wild-type (+) and atm-1 loss-of-function (-) animals with indicated RNAi treatments. Each data point represents the median TOM20 peak number from the plot profile of mitochondrial morphology in a muscle cell. n = 10 per group. Mean ± s.d. *P < 0.05, n.s., not significant. (E) Mitochondrial lengths in body wall muscles in wild-type (+) and atm-1 loss-of-function (-) animals with indicated RNAi treatments. Each data point represents the mitochondrial length measured from an animal body wall muscle cell. n = 20–30 per group. Median with 95% C. I. Mann-Whitney test. *P < 0.05, n.s., not significant. (TIF) [file pgen.1011678.s008.tif]
